# Blood Pressure at Different Life Stages Over the Early Life Course and Intima-Media Thickness

**DOI:** 10.1001/jamapediatrics.2023.5351

**Published:** 2023-12-04

**Authors:** Yaxing Meng, James E. Sharman, Juhani S. Koskinen, Markus Juonala, Jorma S. A. Viikari, Marie-Jeanne Buscot, Feitong Wu, Brooklyn J. Fraser, Suvi P. Rovio, Mika Kähönen, Tapani Rönnemaa, Antti Jula, Harri Niinikoski, Olli T. Raitakari, Katja Pahkala, Costan G. Magnussen

**Affiliations:** 1 Baker Heart and Diabetes Institute, Melbourne, Victoria, Australia; 2 Menzies Institute for Medical Research, University of Tasmania, Hobart, Tasmania, Australia; 3 Baker Department of Cardiometabolic Health, Faculty of Medicine, Dentistry and Health Sciences, University of Melbourne, Melbourne, Victoria, Australia; 4 Research Centre of Applied and Preventive Cardiovascular Medicine, University of Turku, Turku, Finland; 5 Centre for Population Health Research, University of Turku and Turku University Hospital, Turku, Finland; 6 Division of Medicine, Turku University Hospital, Turku, Finland; 7 Department of Medicine, Satakunta Central Hospital, Pori, Finland; 8 Department of Medicine, University of Turku, Turku, Finland; 9 Alliance for Research in Exercise, Nutrition and Activity (ARENA), University of South Australia, Adelaide, South Australia, Australia; 10 Department of Clinical Physiology, Tampere University Hospital and Faculty of Medicine and Health Technology, Tampere University, Tampere, Finland; 11 Department of Chronic Disease Prevention, Institute for Health and Welfare, Turku, Finland; 12 Department of Pediatrics and Adolescent Medicine, Turku University Hospital, University of Turku, Turku, Finland; 13 Department of Clinical Physiology and Nuclear Medicine, Turku University Hospital, University of Turku, Turku, Finland; 14 Paavo Nurmi Centre, Unit of Health and Physical Activity, University of Turku, Turku, Finland

## Abstract

**Importance:**

Although cardiovascular disease (CVD) begins in early life, the extent to which blood pressure (BP) at different life stages contributes to CVD is unclear.

**Objective:**

To determine the relative contribution of BP at different life stages across the early-life course from infancy to young adulthood with carotid intima-media thickness (IMT).

**Design, setting, and participants:**

The analyses were performed in 2022 using data gathered from July 1989 through January 2018 within the Special Turku Coronary Risk Factor Intervention Project, a randomized, infancy-onset cohort of 534 participants coupled with annual BP (from age 7 months to 20 years), biennial IMT measurements (from ages 13 to 19 years), who were followed up with again at age 26 years.

**Exposures:**

BP measured from infancy (aged 7 to 13 months), preschool (2 to 5 years), childhood (6 to 12 years), adolescence (13 to 17 years), and young adulthood (18 to 26 years).

**Main outcomes and measures:**

Primary outcomes were carotid IMT measured in young adulthood at age 26 years. Bayesian relevant life-course exposure models assessed the relative contribution of BP at each life stage.

**Results:**

Systolic BP at each life stage contributed to the association with young adulthood carotid IMT (infancy: relative weight, 25.3%; 95% credible interval [CrI], 3.6-45.8; preschool childhood: relative weight, 27.0%; 95% CrI, 3.3-57.1; childhood: relative weight, 18.0%; 95% CrI, 0.5-40.0; adolescence: relative weight, 13.5%; 95% CrI, 0.4-37.1; and young adulthood: relative weight, 16.2%; 95% CrI, 1.6-46.1). A 1-SD (at single life-stage) higher systolic BP accumulated across the life course was associated with a higher carotid IMT (0.02 mm; 95% CrI, 0.01-0.03). The findings for carotid IMT were replicated in the Cardiovascular Risk in Young Finns Study that assessed systolic BP from childhood and carotid IMT in adulthood (33 to 45 years).

**Conclusion and relevance:**

In this cohort study, a life-course approach indicated that accumulation of risk exposure to BP levels at all life stages contributed to adulthood carotid IMT. Of those, the contribution attributed to each observed life stage was approximately equal. These results support prevention efforts that achieve and maintain normal BP levels across the life course, starting in infancy.

## Key Points

**Question:**

How is blood pressure (BP) across the early life course associated with carotid intima-media thickness in young adulthood?

**Findings:**

In this cohort study, systolic BP from infancy and accumulated across the early life-course was associated with higher carotid intima-media thickness in young adulthood with approximately equal contribution from BP in infancy, preschool childhood, childhood, adolescence, and young adulthood. The cumulative nature of the association was replicated in an independent cohort that assessed BP from childhood to mid-adulthood.

**Meaning:**

Reducing the risk of arterial thickening, and by inference cardiovascular diseases, potentially could be optimized by maintaining lower BP levels since infancy, with each life stage being equally important.

## Teaser

This study attempts to determine the relative contribution of blood pressure at different life stages across the early-life course from infancy to young adulthood with carotid intima-media thickness.

## Introduction

High blood pressure (BP) is a key risk factor for cardiovascular disease (CVD), making it a primary target for intervention in adulthood.^1^ However, the value of screening and treating BP in the pediatric setting is controversial.^2,3,4,5^ Some authorities, including the American Academy of Paediatrics,^2^ the European Society of Hypertension,^3^ and the American Heart Association,^4^ support pediatric BP screening to prevent CVD. But the US Preventive Services Task Force disagrees, citing a lack of evidence to assess the net benefits and harms of screening.^5^ The discrepancy between authorities on the value of screening and treatment for high BP in the pediatric setting highlights the need for greater understanding of the role of BP throughout the life course with CVD. Although observational studies have linked childhood BP to preclinical CVD in adults,^6,7,8^ as well as fatal and nonfatal cardiovascular events,^9^ it remains unclear whether the timing of exposure to heightened BP matters in shaping this association.

Carotid intima-media thickness (cIMT) indicates early vascular remodeling^10,11^ and can predict future CVD.^12^ Research shows that BP in childhood or adolescence, as well as cumulative exposure from childhood to adulthood, are associated with adult cIMT.^7,13,14,15,16,17^ However, these studies have limitations, such as considering BP measured at a single time point in youth or not examining the contribution of BP at different or very early life stages. To gain insight into the role of BP on early arterial injury, and therefore CVD, we aimed to identify how BP measured at different life stages (infancy, preschool childhood, childhood, adolescence, and young adulthood) contributes to cIMT in young adulthood. We also aimed to replicate our findings in a separate child to mid-adulthood cohort study.

## Methods

Details of the study population, BP, cIMT, and covariate measurement, and statistical analyses are provided in Supplement 1 (eMethods 1, eMethods 2, eTable 1, eFigures 1 and 2). This study followed the Strengthening the Reporting of Observational Studies in Epidemiology (STROBE) reporting guidelines.

### Study Design and Participants

Data were from the infancy-onset randomized clinical Special Turku Coronary Risk Factor Intervention Project (STRIP) study, which recruited 5-month-old infants from well-infant clinics in Finland and randomly assigned them to either an intervention or control group.^18^ The intervention group received individualized dietary and subsequently antismoking counseling from the age of 7 months (first measurement time point) to 20 years.^19^ Of those enrolled (n = 1116), 551 attended the follow-up visit at age 26 years, 6 years after the dietary counselling intervention had ceased. This analysis included 534 participants who had BP measured in infancy, preschool childhood, childhood, adolescence, and young adulthood, and who had cIMT at age 26 years. The STRIP study has been conducted according to the guidelines of the Declaration of Helsinki and the study protocol approved by the local ethics committee. Written informed consent was obtained from parents or participants.

### BP Measurements

Trained examiners measured sitting systolic BP (SBP) and diastolic BP (DBP) on the right arm of participants after a 15-minute rest using a oscillometric monitor (Criticon Dinamap 1846 SX until 2001, and Criticon Dinamap Compact T thereafter).^18,20^ BP was standardized as an age-, sex-, and intervention-specific *z*-score to account for the natural variations in BP with age and sex, and potential impact of intervention status.^20^ The same absolute BP values can correspond to different relative positions within the distributions across age, sex, and intervention status (eTable 2 in Supplement 1). Adapting BP values to maintain uniform distribution across various subsets, as the use of *z*-scores allows, offers a uniform metric for comparison by life stage and age point. We calculated life-stage averages for BP using mean values at 7 and 13 months for infancy, 2 to 5 years for preschool childhood, 6 to 12 years for childhood, 13 to 17 years for adolescence, and 18 to 26 years for young adulthood. Pulse pressure was obtained as the difference between SBP and DBP and mean arterial pressure was calculated as DBP plus 40% of pulse pressure.^21^ We focused on SBP, the main determinant of cardiovascular events across all ages,^14^ and provide data for DBP, pulse pressure, and mean arterial pressure in Supplement 1.

### cIMT

Ultrasonography was used to assess IMT of the common carotid artery at ages 11, 13, 15, 17, 19, and 26 years, following standardized protocols (for ages 11 to 19 years: Acuson Sequoia 512; Acuson; for age 26 years: General Electric Vivid E9; GE Vingmend Ultrasound A/S).^18,22,23^ Our analyses primarily focused on cIMT at age 26 years with results from earlier age points provided in the Supplement.

### Covariates

We selected covariates based on previous associations reported between BP and cIMT.^17,20,24,25,26,27,28,29^ Covariates were sex, study group status (control or intervention), height, body mass index, education levels, low-density lipoprotein cholesterol, high-density lipoprotein cholesterol, triglycerides, physical activity, alcohol consumption, smoking, birth weight for gestational age, fasting plasma glucose, parental hypertension history, antihypertension medicine use, infant feeding practices (exclusively breastfed vs mixed), and breastfeeding duration.

### Statistical Analyses

Participant characteristics were examined using Stata version 16.1 (StataCorp). Continuous variables are presented as mean (SD) and categorical variables as proportion and number of participants.

The individual growth curve model (IGC), which quantify changes in BP over time,^30^ was applied to interpolate missing BP values at the individual level.^31,32^ Among enrolled participants, the proportion of those who had SBP measurements at each study age point are shown in eTable 3 in Supplement 1. IGC was performed in R version 3.5.3 (R Foundation for Statistical Computing) using the “lme4” package.

For our primary analyses, the bayesian relevant life-course exposure model (BRLM)^32,33,34,35,36^ was used to assess the relative contribution of BP at life stages: infancy, preschool childhood, childhood, adolescence, and young adulthood with cIMT in young adulthood measured at age 26 years. The BRLM provides an understanding of the long-term association of BP at different life stages on cIMT by determining the life-course hypothesis that best describes the exposure-outcome association. The BRLM estimates the total effect (lifetime effect) of BP across the observed life stages on young adulthood cIMT. If the accumulated BP across the observed life course was associated with the outcome measure, the model would estimate relative weights and their posterior distributions of BP at each life stage with the outcomes. Based on these estimates, the life-course association of BP and cIMT can be conceptualized as an accumulation life-course model (BP levels at each life stage having the same importance), a sensitive life-course model (BP levels at each life stage have a different importance), and the critical life-course model (BP at only 1 life stage is of importance). The shortest Euclidean distance identifies the most optimal life-course hypothesis supported by the data. In the accumulation life-course model, when the relative contribution of each life stage is precisely equal, it is termed a pure accumulation model. However, when the relative contributions are approximately equal, with 95% credible interval (CrI) and posterior distribution overlapping, it is termed a relaxed accumulation model.^35^ Furthermore, the BRLM estimates the life-stage specific effects as the product of the lifetime effect and relative weights, which represents the time dependent association between BP and the outcomes. The “rstan” package of R studio (R Foundation for Statistical Computing) was used to fit the BRLM in the probabilistic programming language, Stan.^37^

## Results

### Participant Characteristics

Participant characteristics are shown in Table 1. Gestational age and birth weight data were retrieved from well baby clinical records for 515 participants, of whom 410 had appropriate birth weight for gestational age (80.0%), 49 were small for gestational age (9.5%), and 56 were large for gestational age (10.8%). Additionally, there was no difference in BP values on average across the entire follow-up period between participants and those lost to follow-up (mean [SD] observed BP incorporating IGC-model derived values: SBP: 107 [6] vs 108 [5] mm Hg; *P* = .10; DBP: 61 [4] vs 61 [3]; *P* = .21; mean [SD] observed BP only: SBP: 105 [7] vs 104 [10] mm Hg; *P* = .06; DBP: 61 [4] vs 60 [6] mm Hg; *P* = .15).

**Table 1.** Characteristics of Participants in Infancy, Preschool Childhood, Childhood, Adolescence, and Young Adulthood

Characteristic	Infancy (7-13 mo)		Preschool (2-5 y)		Childhood (6-12 y)		Adolescence (13-17 y)		Young adulthood (18-26 y)		Lifetime	
	Total No.	Statistic^a^	Total No.	Statistic^a^	Total No.	Statistic^a^	Total No.	Statistic^a^	Total No.	Statistic^a^	Total No.	Statistic^a^
Sex, No. (%)												
Female	299 (56)	299 (56.0)	534	56.0 (299)	534	299 (56.0)	534	299 (56.0)	534	299 (56.0)	NA	NA
Male	235 (44)	235 (44)	534	235 (44)	534	235 (44)	534	235 (44)	534	235 (44)	NA	NA
Intervention, No. (%)	534	254 (47.6)	534	254 (47.6)	534	254 (47.6)	534	254 (47.6)	534	254 (47.6)	NA	NA
Systolic blood pressure, mm Hg,	534	93 (11)	534	99 (7)	534	103 (6)	534	113 (8)	534	120 (10)	534	107 (6)^b^
Diastolic blood pressure, mm Hg	534	60 (9)	534	60 (6)	534	59 (4)	534	61 (5)	534	66 (5)	534	61 (4)^b^
Pulse pressure, mm Hg	534	34 (8)	534	39 (5)	534	45 (5)	534	53 (7)	534	54 (8)	534	45 (5)^b^
Mean arterial pressure, mm Hg	534	73 (9)	534	75 (6)	534	76 (4)	534	82 (5)	534	88 (6)	534	79 (4)^b^
Systolic blood pressure classifications, No. (%)^c^												
Normal	NA	433 (81)	NA	464 (86)	NA	504 (94)	NA	428 (80)	NA	280 (52)	NA	NA
Elevated	NA	42 (7)	NA	37 (7)	NA	19 (3)	NA	83 (15)	NA	170 (31)	NA	NA
Hypertension	NA	59 (11)	NA	33 (6)	NA	11 (2)	NA	23 (4)	NA	84 (15)	NA	NA
Diastolic blood pressure classifications, No. (%)^c^												
Normal	NA	106 (20)	NA	385 (73)	NA	533 (99)	NA	532 (99)	NA	527 (98)	NA	NA
Elevated	NA	88 (16)	NA	93 (17)	NA	1 (0.2)	NA	0	NA	0	NA	NA
Hypertension	NA	340 (67)	NA	56 10)	NA	0	NA	2 (0.1)	NA	7 (1)	NA	NA
Blood pressure classifications, No. (%)^c^												
Normal	NA	105 (20)	NA	357 (67)	NA	503 (94)	NA	428 (80)	NA	280 (52)	NA	NA
Elevated	NA	87 (16)	NA	109 (20)	NA	20 (4)	NA	83 (15)	NA	168 (31)	NA	NA
Hypertension	NA	342 (64)	NA	68 (12)	NA	11 (2)	NA	23 (4)	NA	86 (16)	NA	NA
Height, cm	532	73.8 (2.6)	488	99.6 (4.5)	438	133.9 (7.5)	381	167.6 (7.6)	534	173.2 (9.1)	534	130.0 (15.0)^b^
Body mass index^d^	532	17.1 (1.3)	488	15.9 (1.2)	438	16.8 (2.1)	381	20.4 (2.9)	534	23.4 (4.0)	534	18.5 (2.1)^b^
Non-HDL-C, mmol/L	511	3.32 (0.80)	484	3.35 (0.72)	413	3.23 (0.67)	382	2.91 (0.61)	534	3.13 (0.74)	534	3.20 (0.65)^b^
LDL-C, mmol/L	NA	NA	411	2.97 (0.72)	413	2.87 (0.63)	382	2.50 (0.57)	534	2.66 (0.68)	534	2.73 (0.62)^b^
HDL-C, mmol/L	511	0.91 (0.18)	484	1.11 (0.19)	413	1.31 (0.23)	382	1.18 (0.22)	534	1.33 (0.29)	534	1.18 (0.21)^b^
Triglycerides, mmol/L	NA	NA	411	0.66 (0.25)	413	0.75 (0.29)	382	0.88 (0.33)	534	1.04 (0.41)	534	0.89 (0.35)^b^
Serum glucose, mmol/L	NA	NA	NA	NA	133	4.60 (0.76)	375	4.94 (0.45)	534	4.95 (0.49)	534	4.93 (0.42)^b^
Leisure-time physical activity, MET h/wk	NA	NA	NA	NA	NA	NA	381	27.2 (18.4)	515	24.4 (18.2)	525	26.0 (17.0)^b^
Alcohol consumption, units/wk^e^	NA	NA	NA	NA	NA	NA	247	4.99 (2.96)	356	5.59 (3.65)	361	5.18 (2.93)^b^
Breastfeeding duration, mo^f^	NA	NA	NA	NA	NA	NA	NA	NA	NA	NA	524	7.1 (3.8)
Smoker, No. (%)^g^	NA	NA	NA	NA	NA	NA	NA	24 (7)	NA	81 (16)	NA	85 (16.2)
Highest education level, No. (%)^h^												
Comprehensive or high school	NA	NA	NA	NA	NA	135 (28.5)	NA	NA	NA	166 (32.9)	NA	NA
Bachelor or university	NA	NA	NA	NA	NA	168 (35.5)	NA	NA	NA	284 (56.4)	NA	NA
Master, licentiate, or doctorate	NA	NA	NA	NA	NA	170 (35.9)	NA	NA	NA	54 (10.7)	NA	NA
Anti-hypertensive medication use, No. (%)^i^	NA	NA	NA	NA	NA	NA	NA	NA	NA	7 (1.3)	NA	NA
Parental hypertension, No. (%)^j^	NA	138 (30)	NA	140 (31)	NA	138 (32)	NA	121 (34)	NA	155 (34)	NA	156 (30)
Carotid intima-media thickness, mm^i^	NA	NA	NA	NA	NA	NA	NA	NA	534	0.46 (0.05)	NA	NA

Abbreviations: HDL-C, high-density lipoprotein cholesterol; LDL-C, low-density lipoprotein cholesterol; MET, metabolic equivalent; NA, not applicable.

SI conversion factors: To convert high-density lipoprotein cholesterol to mg/dL, multiply by .0259; low-density lipoprotein cholesterol to mg/dL, multiply by .0259; triglycerides to mg/dL, multiply by .0113; serum glucose to mg/dL, multiply by .0555.

^a^ Data are mean (SD) for continuous variables and proportion (number of participants) for categorical variables.

^b^ The average of values of all life stages for each continuous variable.

^c^ For infancy, preschool childhood, and childhood, classifications were defined according to the 2017 American Academy of Pediatrics guidelines. For adolescence and young adulthood, classifications were defined according to the 2018 American Heart Association guidelines were applied. For the adolescence and young adulthood stages, the categories for elevated and normal diastolic blood pressure were combined, since both were defined a diastolic blood pressure less than 80 mm Hg.

^d^ Calculated as weight in kilograms divided by height in meters squared.

^e^ One unit is approximately 12 grams of alcohol.

^f^ Recorded at 8, 13, 18, 24, 30, and 36 months. Duration of breastfeeding was summed as the months in which breastmilk was the only milk source.

^g^ Regular smoking habits (smoking at least once per day) were reported by participants from age 13 to 26 years. Participants who reported ever smoking once per day or more often in any of the follow-up visits were considered smokers.

^h^ Data on parental education levels were collected on both parents when participants were aged 13 months, 5 years, and 9 years. Participants reported their own education levels at follow-up at age 20 and 26 years.

^i^ Measured at age 26 years follow-up visit.

^j^ Parents were categorized as having hypertension if they self-reported having hypertension or being on medication for hypertension, or if their systolic/diastolic blood pressure were 140/90 mm Hg or higher. One or more parents having hypertension was used to indicate history of parental hypertension. At each life stage, hypertension was defined using mean blood pressure values, calculated from multiple age-points within that stage. For life-course hypertension, mean blood pressure values were averaged across all studied life stages and used to define hypertension.

### Life-Course SBP and IMT in Young Adulthood

Table 2 shows the association of SBP from the 5 life stages with adult cIMT. As reflected by the accumulated estimates, higher cumulative SBP across the observed life course was associated with higher cIMT, independent of adjustment (models 1 and 2; Table 2). The Figure shows relative weights of the association of SBP from the 5 life stages with cIMT in young adulthood. SBP from each life stage contributed to cIMT in young adulthood and the degree to which exposure to SBP associated with cIMT was largely similar across the observed life stages (ranging from 13.5% to 27.0%) (model 2 in the Figure). As the relative weights were not exactly equal with 95% CrI (Figure) and the posterior probabilities of relative weights overlapping (eFigure 3 in Supplement 1) the association between life course SBP and cIMT was suggestive of a relaxed accumulation life-course model. These findings were consistent with the age-specific analyses, indicating that the contribution of SBP was present at the age of 7 months and remained across all observed ages (eTable 4 and eFigure 4 in Supplement 1).

**Table 2.** Association of Systolic Blood Pressure in Infancy, Preschool Childhood, Childhood, Adolescence, and Young Adulthood With Carotid Intima-Media Thickness in Young Adulthood

Life-stage specific effect	Carotid intima-media thickness, β (95% CrI), mm^a^	
	Model 1 (n = 534)^b^	Model 2 (n = 504)^b^
Infancy	0.005 (0.001-0.01)	0.005 (0.0005-0.01)
Preschool childhood	0.006 (0.001-0.01)	0.005 (0.0004-0.01)
Childhood	0.003 (0.0001-0.01)	0.003 (0.0002-0.01)
Adolescence	0.003 (0.0001-0.01)	0.002 (0.0001-0.01)
Young adulthood	0.004 (0.0003-0.01)	0.003 (0.0002-0.01)
Lifetime effect	0.02 (0.01-0.03)	0.02 (0.01-0.03)

Abbreviation: CrI, credible interval.

^a^ β Represents regression coefficient per 1-SD (at single life stage) higher systolic blood pressure level in each life stage (infancy, preschool childhood, childhood, adolescence and young adulthood) and the sum of these across the entire observed life course (lifetime effect).

^b^ Model 1 adjusted for intervention group, sex, and height. Model 2 adjusted for intervention group, sex, height, smoking, body mass index, education levels, low-density lipoprotein cholesterol, high-density lipoprotein cholesterol, triglycerides, physical activity, and birth weight for gestational age. For continuous covariates collected at each time point (height, body mass index, education levels, low-density lipoprotein cholesterol, high-density lipoprotein cholesterol, triglycerides, physical activity), a numerical average from across the life course was used.

**Figure.**
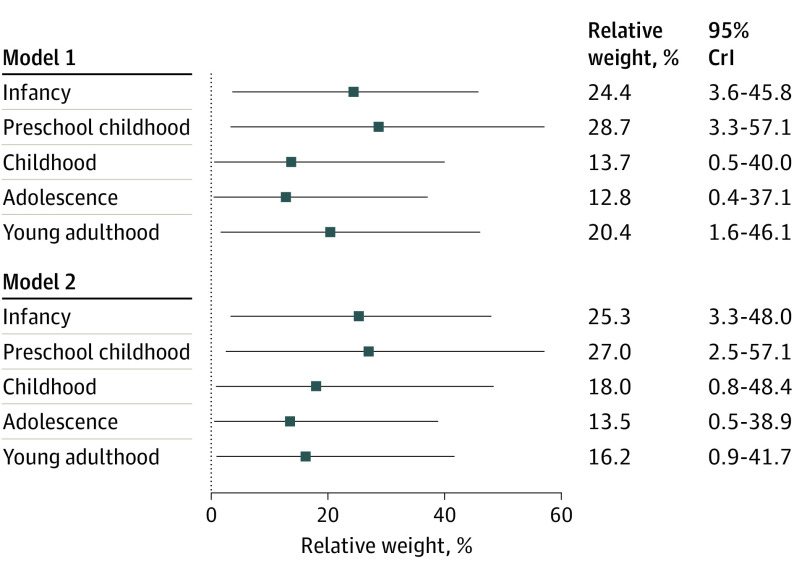
Relative Weights and 95% Credible Intervals (CrIs) of the Association of Systolic Blood Pressure in Infancy, Preschool Childhood, Childhood, Adolescence, and Young Adulthood With Carotid Intima-Media Thickness in Young Adulthood Model 1 is adjusted for intervention group, sex, and height. Model 2 is adjusted for intervention group, sex, height, smoking, body mass index, education levels, low-density lipoprotein cholesterol, high-density lipoprotein cholesterol, triglycerides, physical activity, and birth weight for gestational age.

Results using SBP as a categorical exposure are presented in Table 3. Elevated or hypertensive SBP from infancy to young adulthood was associated with a 0.05 mm (95% CrI, 0.03-0.07) higher cIMT in young adulthood with exposure at each life stage contributing approximately equally. As the severity of SBP increased, the lifetime effect on cIMT increased (hypertensive SBP: β = 0.06; 95% CrI, 0.03-0.10 and elevated SBP: β = 0.04; 95% CrI, 0.01-0.08). When both SBP and DBP were considered for BP classification, hypertension in each observed life stage was equally associated with cIMT, whereas no such association was observed for elevated BP.

**Table 3.** Association of Blood Pressure (BP) Classifications in Infancy, Preschool Childhood, Childhood, Adolescence, and Young Adulthood With Carotid and Aortic Intima-Media Thickness in Young Adulthood^a^

	Systolic BP		Diastolic BP	BP	
	β (95% CrI), mm	Relative weight, % (95% CrI)	β, mm (95% CrI)	β, mm (95% CrI)	Relative weight, % (95% CrI)
**Hypertension**					
Lifetime effect	0.06 (0.03-0.1)	NA	0.03 (−0.04 to 0.16)	0.04 (0.01-0.08)	NA
Infancy	NA	22.6 (4.0-45.0)	NA	NA	20.7 (0.8-54.5)
Preschool childhood	NA	24.3 (2.4-51.5)	NA	NA	30.1 (2.6-66.7)
Childhood	NA	15.6 (0.5-43.0)	NA	NA	17.8 (0.9-47.9)
Adolescence	NA	24.1 (1.8-52.3)	NA	NA	15.7 (0.8-36.1)
Young adulthood	NA	13.2 (0.6-34.6)	NA	NA	15.6 (0.7-42.9)
**Hypertension + elevated**					
Lifetime effect	0.05 (0.03-0.07)	NA	NA	0.03 (0.01-0.05)	NA
Infancy	NA	25.1 (4.1-47.8)	NA	NA	25.6 (1.9-60.1)
Preschool childhood	NA	23.5 (2.3-49.2)	NA	NA	35.2 (6.8-70.5)
Childhood	NA	11.2 (0.3-33.4)	NA	NA	16.1 (0.5-46.4)
Adolescence	NA	19.6 (3.9-39.2)	NA	NA	11.8 (0.4-33.2)
Young adulthood	NA	20.6 (4.1-48.4)	NA	NA	11.2 (0.2-24.6)
**Elevated**					
Lifetime effect	0.04 (0.01-0.08)	NA	NA	−0.03 (−0.08 to 0.04)	NA
Infancy	NA	19.3 (0.7-53.5)	NA	NA	NA
Preschool childhood	NA	16.2 (0.6-46.7)	NA	NA	NA
Childhood	NA	16.0 (0.7-53.5)	NA	NA	NA
Adolescence	NA	23.7 (1.4-57.4)	NA	NA	NA
Young adulthood	NA	24.6 (2.3-56.9)	NA	NA	NA

Abbreviations: CrI, credible interval; NA, not applicable.

^a^ All models adjusted for adjusted for intervention group, sex, height, smoking, body mass index, education levels, low-density lipoprotein cholesterol, high-density lipoprotein cholesterol, triglycerides, physical activity, and birth weight for gestational age. For continuous covariates collected at each time point (height, body mass index, education levels, low-density lipoprotein cholesterol, high-density lipoprotein cholesterol, triglycerides, physical activity), a numerical average from across the life course was used.

Sensitivity analyses were conducted to consider additional potential confounders (eTables 5-7 in Supplement 1) to evaluate the differences between sexes (eTable 8 in Supplement 1), which did not essentially change the findings. Results of estimates using a per 10-mm Hg increment in SBP are presented in eTable 9 in Supplement 1 for BP at specific life stages and eTable 4 in Supplement 1 for BP at individual age points. The threshold change of 10 mm Hg was determined on the basis that it was close to 1 SD of SBP at individual age points (eTable 2 in Supplement 1). The lifetime effects (β = 0.02; 95% CrI, 0.01-0.03) were not markedly different to those in the primary analyses and SBP at each life stage remained approximately equally associated with cIMT in young adulthood (eTable 9 in Supplement 1), which was confirmed by the shortest Euclidian distance (eFigure 1 in Supplement 1). The study team refitted the BRLMs in an independent population-based cohort, the Cardiovascular Risk in Young Finns Study (study population and methodology provided in eMethods in Supplement 1), to examine the association of SBP measured at multiple age points (6 to 45 years at 3-year intervals) and mid-adulthood cIMT. The relative weights of BP at each observed age point had approximately equal contribution to mid-adulthood cIMT with 95% CrI of relative weights overlapping (eFigure 5 in Supplement 1).

DBP (eTable 10 in Supplement 1) or hypertensive DBP (Table 3) was not associated with young adulthood cIMT. The results for pulse pressure (eTables 10-11 in Supplement 1) were similar to the results for SBP. Cumulative mean arterial pressure was associated with cIMT measured at 26 years but was not associated with cIMT at other ages (11 to 19 years) (eTables 10-11 in Supplement 1).

## Discussion

Our study suggests a cumulative impact of SBP across early life on young adulthood cIMT, revealing a roughly equivalent contribution from SBP at each life stage examined. This investigation contributes in 2 key ways. First, by adopting a life-stage perspective starting from infancy, we augment the traditional research lens that has predominantly focused on childhood and adolescence. Second, while our findings align with research associating early-life BP with future cIMT, we introduce additional perspectives by examining the potential significance of the timing of BP exposure in this association. In the absence of life-term clinical trials, our observations emphasize that infancy, preschool childhood, childhood, adolescence, and young adulthood contribute equally to vascular thickening, highlighting the necessity for targeted prevention at all these life stages. This extended view informs our understanding of the developmental origins of cardiovascular health and emphasizes a consistent life-course approach to BP management starting from infancy.^38^

Previous studies have shown that SBP in childhood and adolescence and its cumulative burden are associated with higher cIMT in adulthood.^7,13,14,15,17,39,40^ For example, in the Cardiovascular Risk in Young Finns Study,^17^ SBP measured from age 12 to 18 years was associated with cIMT measured 21 years later after adjusting for current SBP levels. The International Childhood Cardiovascular Cohort Consortium^15^ found a residual association between elevated BP in childhood and adolescence with adulthood cIMT, even among those who achieved normal BP levels in adulthood. Our work extends these studies by examining BP in earlier life stages, such as infancy and preschool. Moreover, we discern the contribution of different life stages of BP exposure, providing a more comprehensive understanding of its association with cIMT. Increased cIMT in adolescence and young adulthood may represent nonatherosclerotic adaptation occurring between lumen diameter and cIMT via the maintenance of local wall shear stress,^39^ although cIMT later in life is a surrogate for atherosclerosis. Nevertheless, in an independent cohort, the association of SBP from childhood to mid-adulthood with mid-adulthood cIMT (eFigure 5 in Supplement 1) was best described by a relaxed accumulated life-course model, replicating our main findings, and lending support to the premise that BP across the early-life course might be involved in atherosclerosis-related remodeling. Ideally, we would confirm this with data on plaque burden, but case numbers were few in both cohorts.

This study has potential implications for CVD prevention by demonstrating the direct and equal contribution of BP at each observed life stage from infancy to cIMT in young adulthood. Interestingly, we found that SBP in early life does not contribute less to the development of cIMT than SBP in adulthood. These findings suggest BP prevention or intervention initiated later in life might be insufficient to reduce the associated risk that has accumulated earlier in life, underscoring the importance of primordial prevention of CVD, as emphasized by the American Heart Association^4^ and others.^38^ Our findings not only reinforce the need for BP prevention in childhood and adolescence but also extend these strategies to very early life. They suggest the potential benefits of routine BP screening even before ages 3 years,^2,3^ highlighting the need for future studies to evaluate the feasibility and risks. The consistent associations between elevated/hypertensive SBP at each life stage and cIMT emphasize the potential benefits of interventions targeting elevated/hypertensive SBP since infancy to obtain vascular health. Altogether, our study emphasizes a life-course approach to BP management, advocating for early intervention and continued prevention throughout all life-stages.

Lifestyle interventions in childhood have demonstrated potential to prevent or delay high BP. For example, in the STRIP study, dietary counselling initiated in infancy led to a sustained reduction in SBP,^20^ even 6 years postintervention.^41^ Similar benefits were observed in the Dietary Intervention Study in Children,^42^ which found a 2.3 mm Hg lower SBP in the intervention group 9 years after the intervention ended. Additionally, the Cardiovascular Health in Children and Youth Study^43^ linked 30 minutes of aerobic exercise 3 days a week for 8 weeks to a 3-mm Hg reduction in SBP. Extrapolating these results using the vascular age (cIMT increases 0.0057 [SD, 0.0004] mm per year in healthy young adults) and our findings that a 10 mm Hg higher SBP through life was associated with a 0.02 mm higher cIMT, these intervention effects could translate to a vascular age reduction by approximately 0.6 to 1.8 years.^44^ While this effect may seem small, even minor shifts in the distribution of pediatric BP could profoundly impact hypertension-related disease at the population level.^8^ Emerging insights into hypertension’s fetal programming^45^ open avenues for preventive measures, complemented by maternal nutrition optimization during pregnancy.^46^ Strategies, such as reducing sodium intake in infancy, may also contribute to preventing high BP levels in adolescence.^47^ Our study emphasizes the need for future research to explore the most effective strategies for BP prevention and management across different life stages, including those highlighted in this study.

### Strengths and Limitations

Our study had several strengths, including the use of an infant cohort and annual BP, biological, and lifestyle measurements collected over a long follow-up period spanning important developmental stages. This study also had limitations. Although loss-to-follow-up occurred, participants and nonparticipants were similar in baseline characteristics, except for a higher proportion of females among participants (eTable 12 in Supplement 1). Additionally, we found no difference in BP values on average across the entire follow-up period between participants and those lost to follow-up (mean [SD] observed BP incorporating IGC model derived values: SBP: 107 [6] vs 108 [5] mm Hg; *P* = .10; DBP: 61 [4] vs 61 [3]; *P* = .21] and mean [SD] observed BP only: SBP: 105 [7] vs 104 [10] mm Hg; *P* = .06; DBP: 61 [4] vs 60 [6] mm Hg; *P* = .15]. Therefore, our loss-to-follow-up comparisons support the notion that those who participated at the last time point are representative of the original sample.^18,41^ Moreover, we took steps to mitigate sample reduction due to missing data, including the use of IGC to interpolate BP. Taken together, we believe that our findings are unlikely to be impacted by differential loss-to-follow-up. The BRLM used does not permit time-varying covariates, so lifetime-averaged values were used instead. However, we observed similar results using this or an approach that analyzed residuals of the primary exposure variable (eTable 13 in Supplement 1). Our primary and replication cohorts were comprised predominantly of White European participants, potentially limiting the generalizability of our results. The oscillometric BP monitor was used throughout the study with BP a single reading at each visit up to age 7 years, potentially impacting the precision of these estimates. Nevertheless, Dinamap’s BP measurement technology has passed validation criteria for the Association for the Advancement of Medical Instrumentation Standard.^48,49,50,51^ This method is different from some pediatric guidelines that recommend using auscultatory BP to confirm high BP from oscillometric measurements.^2,3^ Our study used a standardized BP measurement protocol,^18^ with BP taken by trained staff after a 15-minute rest period for participants, which effectively minimized factors that might artificially elevate SBP. Our participants were part of a dietary counselling and nonsmoking education intervention, which could have influenced their health behaviors. Our study did not include measures of fat and lean muscle, which could be necessary to discern if changes in cIMT due to SBP represent physiological adaptation or arterial injury.^39^ In the absence of sufficient cardiovascular events among our participants, we used IMT as an indicator of arterial injury and future risk of accelerated CVD.^12^

## Conclusions

Building on the established links between BP in childhood and cIMT in adulthood, our study uniquely demonstrates that the relative contribution of BP to cIMT is consistent across all observed life stages, including earlier periods, such as infancy and preschool. These findings suggest that achieving lower BP levels from very early life and maintaining them throughout potentially can reduce the risk of arterial thickening, and by inference, CVD.

## Notes

Supplement 1. eMethods 1. Study design, methods for measuring blood pressure, intima media thickness, and covariates in the Special Turku Coronary Risk Factor Intervention Project eMethods 2. Statistical Analyses eMethods 3. Study design, methods for measuring blood pressure, intima media thickness, and covariates in the Cardiovascular Risk in Young Finns Study eTable 1. Definitions of blood pressure classifications eTable 2. Systolic blood pressure levels at each age point, by intervention and sex eTable 3. Proportion, mean, and standard deviation of systolic blood pressure levels observed and deriveda eTable 4. Relative weights of systolic blood pressure measured at each individual age from 7 months to 26 years and carotid intima-media thickness measured at age of 26 years eTable 5. Association of systolic blood pressure measured in infancy, preschool childhood, childhood, adolescence, and young adulthood and carotid intima-media thickness in young adulthood eTable 6. Association of systolic blood pressure measured in infancy, preschool childhood, childhood, adolescence, and young adulthood and carotid intima-media thickness in young adulthood eTable 7. Association of systolic blood pressure measured in infancy, preschool childhood, childhood, adolescence, and young adulthood and carotid intima-media thickness in young adulthood eTable 8. Association of systolic blood pressure measured in infancy, preschool childhood, childhood, adolescence, and young adulthood and carotid intima-media thickness in young adulthood, stratified by sex eTable 9. Association of systolic blood pressure in infancy, preschool childhood, childhood, adolescence and young adulthood with carotid intima-media thickness in young adulthood eTable 10. Association of diastolic blood pressure, pulse pressure and mean arterial pressure measured in infancy, preschool childhood, childhood, adolescence, and young adulthood and carotid intima-media thickness in young adulthood eTable 11. Association of systolic blood pressure, mean arterial pressure, and pulse pressurea measured from age 7 months up until the age at which carotid intima-media thickness was measured eTable 12. Baseline (age 7 months) characteristics of participants and non-participants at the age 26 years follow-up eTable 13. Association of systolic blood pressure measured in infancy, preschool childhood, childhood, adolescence, and young adulthood and carotid intima-media thickness in young adulthood eTable 14. Systolic blood pressure at each observed time-point in the Cardiovascular Risk in Young Finns Study eFigure 1. Median and 80% credible intervals of posterior distributions of Euclidean distance under three life-course scenarios for the association of systolic blood pressure and carotid intima-media thickness eFigure 2. Diagnostics of the Bayesian relevant life-course exposure model, including (A) trace plots, (B) Rhat values, (C) effective sample size, (D) autocorrelation function plots, (E) identifiability of the parameters, for systolic blood pressure and carotid intima-media thickness eFigure 3. Posterior densities of relative weights for exposure to systolic blood pressure in infancy, preschool childhood, childhood, adolescence, and young adulthood and carotid intima-media thickness in young adulthood eFigure 4. Relative weights of systolic blood pressure measured across each age prior to, and concurrent with, the age when carotid intima-media thickness was measured eFigure 5. Association of systolic blood pressure measured at multiple age points from 0 (aged 33 to 45 years) to 27 years (aged 6 to 18 years) prior to carotid intima media thickness measured in mid-adulthood (aged 33 to 45 years), using data from The Cardiovascular Risk in Young Finns Study

Supplement 2. Data sharing statement
