# Supplementary material for: Blood Pressure at Different Life Stages Over the Early Life Course and Intima-Media Thickness
Source: JAMA Pediatr. 2023 Dec 4;178(2):133–41. doi: 10.1001/jamapediatrics.2023.5351 (PMC10696511; doi:10.1001/jamapediatrics.2023.5351)
Supplement: Supplement 1. — eMethods 1. Study design, methods for measuring blood pressure, intima media thickness, and covariates in the Special Turku Coronary Risk Factor Intervention Project eMethods 2. Statistical Analyses eMethods 3. Study design, methods for measuring blood pressure, intima media thickness, and covariates in the Cardiovascular Risk in Young Finns Study eTable 1. Definitions of blood pressure classifications eTable 2. Systolic blood pressure levels at each age point, by intervention and sex eTable 3. Proportion, mean, and standard deviation of systolic blood pressure levels observed and deriveda eTable 4. Relative weights of systolic blood pressure measured at each individual age from 7 months to 26 years and carotid intima-media thickness measured at age of 26 years eTable 5. Association of systolic blood pressure measured in infancy, preschool childhood, childhood, adolescence, and young adulthood and carotid intima-media thickness in young adulthood eTable 6. Association of systolic blood pressure measured in infancy, preschool childhood, childhood, adolescence, and young adulthood and carotid intima-media thickness in young adulthood eTable 7. Association of systolic blood pressure measured in infancy, preschool childhood, childhood, adolescence, and young adulthood and carotid intima-media thickness in young adulthood eTable 8. Association of systolic blood pressure measured in infancy, preschool childhood, childhood, adolescence, and young adulthood and carotid intima-media thickness in young adulthood, stratified by sex eTable 9. Association of systolic blood pressure in infancy, preschool childhood, childhood, adolescence and young adulthood with carotid intima-media thickness in young adulthood eTable 10. Association of diastolic blood pressure, pulse pressure and mean arterial pressure measured in infancy, preschool childhood, childhood, adolescence, and young adulthood and carotid intima-media thickness in young adulthood eTable 11. Association of systolic bloo [file jamapediatr-e235351-s001.pdf]

Supplemental Online Content

Meng Y, Sharman JE, Koskinen JS, et al. Blood pressure at different life-stages over the early life-course and intima-media thickness. *JAMA Pediatr*. Published online December 4, 2023. doi:10.1001/jamapediatrics.2023.5351

eMethods 1. Study design, methods for measuring blood pressure, intima media thickness, and covariates in the Special Turku Coronary Risk Factor Intervention Project

eMethods 2. Statistical Analyses

eMethods 3. Study design, methods for measuring blood pressure, intima media thickness, and covariates in the Cardiovascular Risk in Young Finns Study

eTable 1. Definitions of blood pressure classifications

eTable 2. Systolic blood pressure levels at each age point, by intervention and sex

eTable 3. Proportion, mean, and standard deviation of systolic blood pressure levels observed and derived[a]

eTable 4. Relative weights of systolic blood pressure measured at each individual age from 7 months to 26 years and carotid intima-media thickness measured at age of 26 years

eTable 5. Association of systolic blood pressure measured in infancy, preschool childhood, childhood, adolescence, and young adulthood and carotid intima-media thickness in young adulthood

eTable 6. Association of systolic blood pressure measured in infancy, preschool childhood, childhood, adolescence, and young adulthood and carotid intima-media thickness in young adulthood

eTable 7. Association of systolic blood pressure measured in infancy, preschool childhood, childhood, adolescence, and young adulthood and carotid intima-media thickness in young adulthood

eTable 8. Association of systolic blood pressure measured in infancy, preschool childhood, childhood, adolescence, and young adulthood and carotid intima-media thickness in young adulthood, stratified by sex

eTable 9. Association of systolic blood pressure in infancy, preschool childhood, childhood, adolescence and young adulthood with carotid intima-media thickness in young adulthood

eTable 10. Association of diastolic blood pressure, pulse pressure and mean arterial pressure measured in infancy, preschool childhood, childhood, adolescence, and young adulthood and carotid intima-media thickness in young adulthood

eTable 11. Association of systolic blood pressure, mean arterial pressure, and pulse pressure[a] measured from age 7 months up until the age at which carotid intima-media thickness was measured

eTable 12. Baseline (age 7 months) characteristics of participants and non-participants at the age 26 years follow-up

eTable 13. Association of systolic blood pressure measured in infancy, preschool childhood, childhood, adolescence, and young adulthood and carotid intima-media thickness in young adulthood

eTable 14. Systolic blood pressure at each observed time-point in the Cardiovascular Risk in Young Finns Study

eFigure 1. Median and 80% credible intervals of posterior distributions of Euclidean distance under three life-course scenarios for the association of systolic blood pressure and carotid intima-media thickness

© 2023 Meng Y et al. *JAMA Pediatrics.*

eFigure 2. Diagnostics of the Bayesian relevant life-course exposure model, including (A) trace plots, (B) Rhat values, (C) effective sample size, (D) autocorrelation function plots, (E) identifiability of the parameters, for systolic blood pressure and carotid intima-media thickness

eFigure 3. Posterior densities of relative weights for exposure to systolic blood pressure in infancy, preschool childhood, childhood, adolescence, and young adulthood and carotid intima-media thickness in young adulthood

eFigure 4. Relative weights of systolic blood pressure measured across each age prior to, and concurrent with, the age when carotid intima-media thickness was measured

eFigure 5. Association of systolic blood pressure measured at multiple age points from 0 (aged 33 to 45 years) to 27 years (aged 6 to 18 years) prior to carotid intima media thickness measured in mid-adulthood (aged 33 to 45 years), using data from The Cardiovascular Risk in Young Finns Study

This supplemental material has been provided by the authors to give readers additional information about their work.

© 2023 Meng Y et al. *JAMA Pediatrics.*

eMethods

eMethods1: Study design, methods for measuring blood pressure, intima media thickness, and covariates in the Special Turku Coronary Risk Factor Intervention Project

Study design and participants

Participants of this study were from the ongoing STRIP, which is an infancy-onset randomized controlled trial of dietary counselling performed for two decades until adulthood that aimed to reduce the risk of atherosclerosis.[1] In brief, the families of five-month-old infants born between July 1989 and December 1991 were recruited at well-baby clinics in Turku, Finland by nurses. At age seven months, 1062 infants (56.5% of those eligible) were randomly allocated to intervention (n=540) or control (n=522) groups.[2] Additionally, the cohort included two children with Down syndrome, two with familial hypercholesterolemia, and five children randomized into the intervention group but who had missed the first study appointments prior to age 13 months and were later treated as controls.[2] A group of 45 children, born between March and July 1989, were also similarly recruited and randomized (intervention n=22, control n=23) to first test the study protocols, and thus served as a 'pilot' group.[2] The intervention group received individualized dietary and subsequently antismoking counselling at 1- to 3-month intervals from age 7 months (first measurement time-point) until 2 years and twice per year thereafter until the age of 20 years. Children in the control group attended the study clinic biannually until age 7 years, and annually thereafter until age 20 years. Comparable measurements were performed for both groups with the same study personnel collecting the measurements. Of those enrolled, 551 attended the follow-up at age 26 years, 6 years after the dietary counselling intervention had ceased, and 534 participants included in present analyses. The STRIP has been conducted according to the guidelines of the Declaration of Helsinki, and the study protocol approved by the local ethics committee. Parents provided written informed consent at study entry and until age 15 years, from then participants provided their own written informed consent at each survey from age 15 to 20 years and at the age 26-year follow-up.

Blood pressure measurements

Systolic BP (SBP) and diastolic BP (DBP) of intervention and control children were measured by trained examiners according to a standardized protocol at each survey using a validated oscillometric BP monitor (Criticon Dinamap 1846 SX until 2001, thereafter Criticon Dinamap Compact T).[1,3] The precision of the device was regularly checked against a mercury manometer. Oscillometric devices, such as Dinamap, are commonly used in large-scale epidemiological studies involving very young individuals due to their operational advantages such as ease of use, automation, and minimizing inter-observer variability.[4] In pediatrics and adults, these devices have passed validation criteria according to the Association for the Advancement of Medical Instrumentation Standard (ANSI/AAMI SP-10).[5-8]The challenges of the auscultatory method in hearing Korotkoff sounds or differentiating Korotkoff phases (IV and V) in young children,[4,9] further justify the use of the oscillometric method in this context. In the intervention group, BP was measured biannually from age 7 months until 20 years, and at age 26 years. Participants in the control group had their BP measured biannually from age 7 months to 7 years and annually thereafter until age 20 years, and at age 26 years. This study was restricted to BP measurements recorded at age 7 months, 13 months, then from 1-year intervals from age 2-20 years, and at age 26 years. At each survey, intervention and control group participants were measured by trained examiners in the same environment. BP measurements were taken on the right arm of participants after about 15-minutes rest in the seated position and using an appropriate cuff size. The manufacturer of the BP device provided four cuff sizes, with the appropriate cuff size used according to the participant's right arm circumference: infant size for participants with a limb circumference of 8-13 cm, child size for participants with a limb circumference of 12-19 cm, small adult size for participants with a limb circumference of 17-25 cm, and adult size for participants with a limb circumference of 23-33 cm. BP was measured once at each survey until 7 years of age, thereafter two to four successive BP measurements were taken (observing a 3-minute interval between successive measurements).[3,10]

Blood pressure classifications

We categorized BP as normal, elevated, and hypertensive at each life-stage. Where age was under 13 years, we used the 2017 American Academy of Pedatrics guidelines,[11] aligning BP values at 7 months in our sample with the one-year-old norms. Life-stage averages were computed from mean values at different age ranges: 7 and 13 months for infancy, 2-5 years for preschool childhood, and 6-12 years for childhood. For adolescence (13-17 years) and young adulthood (18-26 years), where age was 13 years or older, classifications were made using the

© 2023 Meng Y et al. *JAMA Pediatrics.*

2018 American College of Cardiology/American Heart Association guidelines.[12] Details can be found in eTable 1.

Our study used the Bayesian relevant life-course exposure model (BRLM), which limits us to binary variables. Therefore, we conducted separate analyses to examine associations between various combinations of the BP classification and carotid intima-media thickness (cIMT).

Carotid intima-media thickness

Participants fasted for at least 4 hours prior to ultrasound imaging and were advised to refrain from a high-fat meal, fresh juice, caffeinated drinks, smoking, and consuming vitamin supplements on the day of the ultrasonography. The ultrasonography studies were performed in silence in a temperature controlled clinical research laboratory. IMT of the common carotid artery were assessed using ultrasonography (at age 11 to 19 years: Acuson Sequoia 512; Siemens Healthcare, Mountain View, CA, USA; at age 26 years: GE Vivid E9; GE Vingmend Ultrasound A/S, Horten, Norway) according to standardized protocols.[13,14] Ultrasound scans were performed by experienced sonographers blinded to participant details.

For assessment of cIMT, both right and left carotid arteries were scanned with a 15 L 8 linear array transducer at ages 11, 13, 15, 17, and 19 years. The posterior far wall of the common carotid artery was scanned from lateral and anterior oblique angles. The carotid bifurcation was identified, and gain settings were used to optimize image quality so that both the intima of the near and far wall of the common carotid artery 10 to 20 mm proximal to the carotid bifurcation was visible. The resolution box function was used to magnify a 20 mm width image of the common carotid artery far wall and end-diastolic (incident with the R wave on a continuously recorded electrocardiogram) frames were captured and stored for subsequent off-line analysis. At the age 26-year follow-up, right and left common carotid arteries were scanned with an 11 L linear transducer. With the carotid bifurcation identified, the image was focused on the common carotid artery 10 mm proximal to the carotid bifurcation so that both the intima of the near and far wall was visible. A moving scan, including the common carotid artery and the beginning of the carotid bifurcation with a duration of 5 seconds was captured and stored for subsequent off-line analysis. At ages 11, 13, 15, 17, and 19 years, cIMT was measured manually using ultrasonic calipers by readers blinded to participant details. Four cIMT measurements were taken from both lateral and anterior oblique angles on the right and left common carotid artery. The mean value of these IMT measurements was used in statistical analysis. At the age 26-year follow-up, cIMT measurements were obtained with a semi-automated TOMTEC AutoIMT (Version TTA2.41.00., TOMTEC Imaging Systems GmbH, Unterschleissheim, Germany) by a single reader blinded to participant details. The best quality end-diastolic (incident with the R wave on a continuously recorded electrocardiogram) frame was selected from the 5-second clip image. On the selected frame, a box approximately 10 mm wide (region of interest) was drawn with TOMTEC 5 to 15 mm proximal to the carotid bifurcation. Then, the program automatically recognized the lumen-intima and media-adventitia boundaries of the far wall, drew lines at the boundaries and automatically calculated the mean IMT for the defined region of interest. Automated IMT measurements were approved by the reader. If the automatic detection of the arterial boundaries was not satisfactory, the reader made three manual IMT measurements. The mean of the right and left cIMT was used for statistical analysis.

Previously in our laboratory, the between-observer coefficients of variation (CV) of cIMT measurements were 3.0%. And the between-visit CV of cIMT measurements were 3.9%.[13,14] To assess between-observer and between-visit reproducibility for the 26-year follow-up, we re-examined 50 subjects (~10% random sample) with TOMTEC. The between-observer CV of cIMT measurements was 5.3%. The between-visit CV of cIMT measurements was 3.8%.

Covariates

An infant board (Bekvil; Paljerakenne, Helsinki, Finland) was used to measure recumbent length of participants when aged <2 years; with standing height measured thereafter to 0.1 cm using a Harpender stadiometer (Holtain, Crymych, United Kingdom).[15] Weight was measured to 0.1 kg using an infant scale (Seca 725; Hamburg, Germany) until age 15 months, with an electronic scale (S10; Soehnle, Murrhardt, Germany) used thereafter.[10] Body mass index was calculated as weight (kg) divided by height (m) squared.

A venous blood sample was drawn at 7 months, 13 months, and 2 years of age and annually thereafter (excluding ages 6 and 8 years).[15] Non-fasting venous blood samples were taken until age 5 years, thereafter fasting blood samples were obtained.[15,16] Serum lipid and apolipoprotein determinations have been described in detail elsewhere.[16] Briefly, serum total cholesterol and triglyceride cholesterol concentrations were measured with a fully enzymatic cholesterol oxidase-p-aminophenazone method (CHOD-PAP; Merck, Darmstadt, Germany) equipped with an automatic Olympus AU400 analyser. Serum high-density lipoprotein cholesterol

© 2023 Meng Y et al. *JAMA Pediatrics.*

(HDL-C) concentration was measured after precipitation of low-density lipoprotein cholesterol (LDL-C) and very low-density lipoprotein cholesterol with dextran sulfate 500000. Serum LDL-C values were calculated using the Friedewald formula.[17] Non-HDL-C was equal to total cholesterol minus HDL-C. At age 7 years, a subsample of 224 participants from the intervention (n=114) and control (n-110) groups had their fasting serum glucose measured, and thereafter at 2-years intervals until age 13 years.[18] From age 15 years onwards, annual fasting glucose was measured from all participants.[18] Serum glucose was measured by the glucose dehydrogenase method (Merck Diagnostica) from age 7 to 13 years and by a hexokinase method (Glucose Olympus System Reagent; Olympus, Ireland) from age 15 years to 20 years[18] and by the enzymatic hexokinase method (glucose reagent, Beckman Coulter) at age 26 years.[15]

Information on participant's parental and own education level was used to indicate socioeconomic status (parental education levels were used for pre-adulthood, and participants own education levels were used for adulthood). Education level was categorized into three classes ranging from 1 to 3 as: (1) comprehensive or high school, (2) bachelor's degree or university courses without a degree, (3) master's degree, licentiate, or doctorate. Data on parental education levels were collected on both parents when participants were aged 13 months, 5 years, and 9 years. The highest education level (either from the mother or the father) in those three surveys was considered as the indicator of earlier-life socioeconomic status. In the case of missing information from either of the parents, the one obtained was used. Similarly, participants reported their own education levels at follow-up at age 20 and 26 years, and the most recent information was used to indicate adulthood socioeconomic status. To compare the education levels at different periods, the values were converted to survey-specific Z-scores.

Lifestyle information, including physical activity,[19,20] smoking,[19] and alcohol consumption were collected by questionnaires. Leisure-time physical activity comprising recreational and organized physical activity/sports outside school hours was assessed at the ages of 13 to 19 years with 2-year intervals and again at age 26 years with a self-administered questionnaire in which frequency, duration, and the intensity of leisure-time physical activity was reported.[20] Leisure-time physical activity was calculated as metabolic equivalent (MET), hours per week by multiplying resting metabolic rate by the mean frequency, duration, and intensity of weekly leisure-time physical activity.[20] Regular smoking habits (smoking at least once per day) were reported by participants from age 13 to 26 years. Participants who reported ever smoking once per day or more often in any of the follow-up visits were considered smokers. Participants reported their alcohol consumption during the last week beginning from age 15 years. Participants were asked to report their consumption of 1/3 l cans or bottles of beer, glasses (12 cl) of wine, and 4 cl shots of liquor or strong alcohol during the last week. These amounts are comparable to approximately 12 g of alcohol (=1 unit). Beverages consumed during the past week were totaled to determine alcohol consumption.

Data on birthweight were collected from well-baby clinic records. Birth weight for gestational age was generated based on Finnish sex and gestational week-stratified birth weight percentiles.[21] Participants were categorized as small birth weight for gestational age if their birth weight for gestational age was less than or equal to the 10th percentile; appropriate birth weight for gestational age if their birth weight for gestational age was more than the 10th percentile and less than or equal to the 90th percentile; large birth weight for gestational age if their birth weight for gestational age was more than the 90th percentile.[21]

Participants were asked "Have you ever used anti-hypertension medicine (yes/no)?" at the age 26 years follow-up.[15] Those who answered "yes" were defined as using anti-hypertension medication.

Parental BP levels of participants were measured between one and four times at each survey using an oscillometric device (Criticon Dinamap 1846 SX until 2001, thereafter Criticon Dinamap Compact T) after rest for about 15 minutes. The mean of all available readings was used. If data were missing from either parent, the one obtained was used. Parents were categorized as having hypertension if they self-reported having hypertension or being on medication for hypertension, or if their systolic BP (SBP) or diastolic BP (DBP) were ≥140 or 90 mmHg, respectively.[22] One or more parents having hypertension was used to indicate history of parental hypertension.

Food consumption of infants and preschoolers was recorded at 8, 13, 18, 24, 30, and 36 months of age. Types of feeding included: exclusive breastfeeding (breastmilk was the only milk source), the combination of breastfeeding and formula, and commercial cow milk–based formulas. Given the BRLM allows only dichotomous variables and not multi-categorical variables, the latter two types were combined in this study. Duration of breastfeeding (in months) was summed as the months in which breastmilk was the only milk source.

© 2023 Meng Y et al. *JAMA Pediatrics.*

eMethods 2: Statistical Analyses

Individual growth curve model

The individual growth curve (IGC)[23] model is an advanced multilevel mixed effect regression that can deal with repeated measurements and different numbers of individual observations at unequal time intervals. The IGC model is used for exploring change in a variable over time containing both inter-individual and intra-individual variability. This technique also allows the quantification of linear or non-linear trajectories over time. In our case, IGC was applied to determine the growth change of BP from age 7 months to 26 years at both the individual level and the group level (i.e., average population level). Then the individual level data derived from the model were used to interpolate over the ages with missing data.

We firstly constructed an unconditional model (i.e., random intercept) where BP is modelled as a function of age, and each participant is used as a random effect. Linear and higher power items of age were added into the model sequentially. When comparing increasingly complex models throughout the IGC analyses, Akaike information criterion (AIC) and likelihood ratio test were used to assess model improvement. If the higher power items were not statistically significant, or if the AIC value of the model was not improved, or if singularity or non-convergence occurred, these higher-order terms were dropped. After the best unconditional model was determined, the model was expanded by adding random slope terms for linear and higher power terms of age. This allowed us to test sequentially if each additional random parameter (i.e., random intercept, random linear slope, random quadratic slope…) improved the fit of the unconditional models. After determining the random intercept and random slope, sex, intervention group and height were introduced into the model to test whether they improve the accuracy for predicting BP changes. In this study, the SBP growth curve was best described by a quartic age (age^4) term and inclusion of intervention, sex and height as modifiers. Our IGC models for DBP, pulse pressure, and mean arterial pressure contained the same quartic age term, intervention, sex, and height as for SBP. The "Lme4" package of R studio (Version 3.5.3, R Foundation for Statistical Computing, Vienna, Austria) was used to perform the IGC modelling.

The Bayesian relevant life-course exposure model

The BRLM considers a model of weighted exposure for each observed life stage, conceptualized as the product of the exposure metric and its corresponding weight over each of the life stages, summed across all life stages.[24-26] The BRLM encompasses the hierarchical structure of life-course models and as such, does not require model or variable selection.[24,25] For our purpose, the BRLM provides an understanding of the long-term association of exposure (BP) at different life stages on the outcomes (cIMT) by determining the life-course hypothesis that best fits the association between exposure and outcome. The BRLM assumes a weight for the exposure variable at each life-stage using a Bayesian approach. The life-stage specific weight parameters relate to the relative importance of exposure at each individual life stage for predicting the development of an outcome later in life. Moreover, the BRLM estimates a total effect for lifetime exposure, that represents the maximum accumulated effect of the exposure variable across the observed life-course on the outcome of interest (lifetime effect). Furthermore, the BRLM method derives life stage specific estimates that combine the lifetime effect and relative weights. Life stage specific effects represent the time dependent association of the exposure variable and the observed outcome measure. There was little or no evidence to include prior beliefs on what life-course model would best support these data. Therefore, a non-informative prior, giving equal support to all life models, was used. In the current study, BRLMs were fitted using a non-informative Dirichlet (1, 1, 1,1,1) prior for weights and a weekly informative Cauchy prior (0, 2.5) for the lifetime effect. In the present five life-stages (infancy, preschool childhood, childhood, adolescence, young adulthood) study, the model assumptions included one accumulation life-course model (all weights = 1/5), four critical life-course models (one of the five weights = 100% and the other four = 0), and one sensitive model (weight in infancy = 2.5%, weight in preschool childhood = 7.5%, weight in childhood = 12.5%, weight in adolescence = 20%, weight in young adulthood = 57.5%).

Because the weights inform the life-course model, they are estimated directly from the data. Once the posterior distribution of weights conditioned on a non-informative prior has been estimated using Bayesian inference, these weight distributions are used to identify the optimal life-course model that is supported by the data by calculating the Euclidean distance, a measure of difference between estimated and expected weight vectors under a fixed number of life-course hypotheses. The life-course hypothesis identified as having the shortest Euclidean distance (eFigure 1) is the model (accumulation, sensitive or critical life-course model) most supported by the data. In our study, the shortest Euclidean distance corresponds to the accumulation life-course model (eFigure 1). In addition, the credible intervals and posterior distribution of estimates were wide and overlapped. Therefore, even though the estimated relative weights of SBP in the earlier life-stages were higher

© 2023 Meng Y et al. *JAMA Pediatrics.*

than those in the later life-stages, such as infancy (25.3%) versus young adulthood (16.2%) (Figure 1), it does not mark the earlier life-stages as more sensitive.

The model diagnosis includes the following aspects. Convergence and mixing were evaluated by means of trace plots and Rhat values, autocorrelation was assessed using autocorrelation function plots, identifiability of the parameters was examined using pairs plots, effectiveness of the sampler was assessed using effective sample size Neff metrics. Diagnostics of the final fitted BRLM model (i.e., model 2) suggested adequate convergence, mixing, and effective sample size and no autocorrelation or identifiability issue (eFigure 2). Results were similar for DBP, pulse pressure and mean arterial pressure (data not shown). In R (Version 3.5.3, R Foundation for Statistical Computing, Vienna, Austria) using the Stan package[27] to fit the BRLMs.

© 2023 Meng Y et al. *JAMA Pediatrics.*

eMethods 3: Study design, methods for measuring blood pressure, intima media thickness, and covariates in the Cardiovascular Risk in Young Finns Study

Study design and participants

The Cardiovascular Risk in Young Finns Study is an ongoing, population-based, 5-center prospective cohort of atherosclerosis risk factors from childhood to adulthood.[28] The first cross-sectional survey was launched in 1980 among 3596 participants aged 3, 6, 9, 12, 15 and 18 years (year of birth: 1977, 1974, 1971, 1968, 1965 and 1962, respectively) who were representative of the national population at that time.[29] Follow-up surveys were conducted in 1983, 1986, 1989, 1992, 2001, 2007, 2011 and 2018. In the current analyses, participants from the youngest birth cohort (aged 3 years in 1980) were not included, because BP for this subgroup was measured by an ultrasound device. The latest follow-up included in the current study was 2007, as the most recent cIMT was measured during this year of follow-up. The study sample included 1865 participants from the 5 oldest cohorts (aged 6 to 18 years in 1980) who had BP measured at least once and had their cIMT measured in 2007 (aged 33 to 45 years). Mean (standard deviation) SBP at each observed time-point was summarized in Table S13. The study received approval by local ethics committees and was conducted according to the Declaration of Helsinki. Participants or their guardians provided written informed consent.

Blood pressure measurements

SBP and DBP levels were measured from the right brachial artery using a standard mercury sphygmomanometer (1980 and 1983), and with a random zero sphygmomanometer (Hawksley & Sons, Lancin, UK) (1986 to 2007).[28,29] The first Korotkoff phase was used to determine SBP, and the fifth phase was used to determine DBP. At all surveys, BP was measured by trained examiners using a standard protocol. After the participants observed a 5-minutes rest, three successive readings (with about 3-minute intervals) were obtained seated using an appropriately sized cuff. The average of three readings was used to represent BP at each survey. Mean (SD) SBP at each observed time-point was summarized in eTable13. Missing SBP values were interpolated via an IGC model.[23,30,31] The systolic BP trajectory in the YFS was best defined by an IGC model with quartic age polynomial (age^4) random intercept, cubic age random slope with sex and height included as modifiers.

Carotid intima-media thickness

cIMT measurements were measured using ultrasound mainframes (Sequoia 512, Acuson, Mountain View, Calif) with 13.0-MHz linear array transducers by experienced ultrasound technicians using a standardized protocol.[32] The image, focused on the posterior (far) wall of the left common carotid artery, included the common carotid artery and carotid bifurcation. The ultrasound image and the off-line analysis of the scans were performed by a single reader blinded to participant data. Gain settings were user-defined to optimize image quality. A region of interest function was used to record an image of 25 mm wide by 15 mm high. At least four end-diastole (determine from concomitant ECG) measurements were recorded at approximately 10 mm proximal to the carotid bifurcation, and the average of these four measurements were used.

The reproducibility of the cIMT image analyses were assessed among 113 participants aged 24 to 39 years. Scans were analyzed by two independent observers blinded to participant details. The between-observer coefficient of variation was 5.2%.[32] To assess intraindividual reproducibility of cIMT measurements, 60 participants were re-examined 3 months after the initial visit. The between-visit coefficient of variation of cIMT measurements was 6.4%.[32]

Covariates

Covariates in the analyses using data from the Cardiovascular Risk in Young Finns Study were year of birth, sex, BMI, height, education levels, fasting plasma glucose, LDL-C, HDL-C, TG, physical activity, alcohol consumption, and smoking. Model 1 included year of birth, sex, and height as covariates. Model 2 additionally adjusted for BMI, education levels, fasting plasma glucose, LDL-C, HDL-C, TG, physical activity, alcohol consumption, and smoking.

At each survey, height and weight were measured[28] and BMI was calculated as weight (kg) divided by height (m) squared. Blood samples were collected from the right antecubital vein after a 12-hour fast.[28] At each study time-point, standard enzymatic methods were used for measuring levels of serum TC, TG and HDL-C.[28] Serum LDL-C values were calculated using the Friedewald formula.[17] Fasting plasma glucose was measured among participants aged 12 years and older beginning from the 1986 survey. Glucose concentrations were determined by the enzymatic hexokinase method.[28] In 2001 and 2007, participants reported their consumption of 1/3 litre cans or bottles of beer, 1/3 litre glasses of wine, and 4 centiliter shots of liquor or strong alcohol during

© 2023 Meng Y et al. *JAMA Pediatrics.*

the past week. These amounts are comparable to approximately 14 g of alcohol (=1 unit).[33] The total consumption of different beverages in the last week was as the total alcohol consumption per week. At each survey, data on physical activity was self-reported by participants aged 9 years and older.[34] In 1980, 1983, 1986, and 1989, the questionnaire included: frequency and intensity of leisure-time physical activity, participation in sports club training, participation in sport competitions, and habitual leisure time.[34] In the follow-ups conducted from 1992 onwards, the physical activity questionnaire included: the frequency and intensity of physical activity, frequency of vigorous physical activity, hours spent on vigorous physical activity, average duration of a physical activity session, and participation in organized physical activity.[34] The items were coded from 1 to 3 and summed to form a physical activity index with scores at each study time-point.[34] At each survey, data on smoking habits were collected among participants aged more than or equal to age 12 years using self-reported questionnaires.[28] Pack-years of smoking was calculated as the number of daily cigarette packs smoked multiplied by daily smoking duration in years. For participants aged 6 to 21 years, completed years of schooling for their parent with the highest education were used.[35] For participants aged 24 years and older, highest level of educational attendance or completed education of themselves was used.[35] Z-scores of education levels were used in the analyses for this study.

© 2023 Meng Y et al. *JAMA Pediatrics.*

References

1. Simell O, Niinikoski H, Rönnemaa T, et al. Cohort Profile: The STRIP Study (Special Turku Coronary Risk Factor Intervention Project), an infancy-onset dietary and life-style intervention trial. *Int J Epidemiol*. 2009;38(3):650-655. doi:10.1093/ije/dyn072
2. Rovio SP, Salo H, Niinikoski H, et al. Dietary intervention in infancy and cognitive function in young adulthood: The Special Turku Coronary Risk Factor Intervention Project. *The Journal of pediatrics*. Mar 30 2022;246:184-190. doi:10.1016/j.jpeds.2022.03.046
3. Niinikoski H, Jula A, Viikari J, et al. Blood pressure is lower in children and adolescents with a low-saturated-fat diet since infancy. *Hypertension*. 2009;53(6):918-924. doi:10.1161/hypertensionaha.109.130146
4. Lim SH, Kim SH. Blood pressure measurements and hypertension in infants, children, and adolescents: from the postmercury to mobile devices. *Clin Exp Pediatr*. Feb 2022;65(2):73-80. doi:10.3345/cep.2021.00143
5. Olukayode AV, Michael Olaniyan O, Oluwatoyin O. Validation Study of the Dinamap Compact TS According to the International Protocol of European Society of Hypertension in Preschool Children in Ogbomoso, Nigeria. *Int. J. Health Res*. 2022;12(4):1-11. doi:10.52403/ijhsr.20220401
6. Frank's Hospital Workshop website. About DINAMAP® Compact Monitor operation manual. http://www.frankshospitalworkshop.com/equipment/documents/ecg/user_manuals/Critikon_Dinamap_Compact_-_User_manual.pdf. Accessed August 16, 2023.
7. Mundt KA, Chambless LE, Burnham CB, Heiss G. Measuring ankle systolic blood pressure: validation of the Dinamap 1846 SX. *Angiology*. 1992;43(7):555-66. doi:10.1177/000331979204300703
8. Park MK, Menard SM. Accuracy of blood pressure measurement by the Dinamap monitor in infants and children. *Pediatrics*. Jun 1987;79(6):907-14.
9. Stergiou GS, Boubouchairopoulou N, Kollias A. Accuracy of Automated Blood Pressure Measurement in Children: Evidence, Issues, and Perspectives. *Hypertension*. 2017;69(6):1000-1006. doi:10.1161/hypertensionaha.116.08553
10. Mikola H, Pahkala K, Niinikoski H, et al. Cardiometabolic determinants of carotid and aortic distensibility from childhood to early adulthood. *Hypertension*. 2017;70(2):452-460. doi:10.1161/hypertensionaha.117.09027
11. Flynn JT, Kaelber DC, Baker-Smith CM, et al. Clinical practice guideline for screening and management of high blood pressure in children and adolescents. *Pediatrics*. 2017;140(3):e20171904. doi:10.1542/peds.2017-1904
12. Grundy SM, Stone NJ, Bailey AL, et al. 2018 AHA/ACC/AACVPR/AAPA/ABC/ACPM/ADA/AGS/APhA/ASPC/NLA/PCNA Guideline on the Management of Blood Cholesterol: A Report of the American College of Cardiology/American Heart Association Task Force on Clinical Practice Guidelines. *Circulation*. 2019;139(25):e1082-e1143. doi:10.1161/cir.0000000000000625
13. Kallio K, Jokinen E, Saarinen M, et al. Arterial intima-media thickness, endothelial function, and apolipoproteins in adolescents frequently exposed to tobacco smoke. *Circ Cardiovasc Qual Outcomes*. 2010;3(2):196-203. doi:10.1161/circoutcomes.109.857771
14. Järvisalo MJ, Jartti L, Näntö-Salonen K, et al. Increased aortic intima-media thickness: A marker of preclinical atherosclerosis in high-risk children. *Circulation*. 2001;104(24):2943-2947. doi:10.1161/hc4901.100522
15. Pahkala K, Laitinen TT, Niinikoski H, et al. Effects of 20-year infancy-onset dietary counselling on cardiometabolic risk factors in the Special Turku Coronary Risk Factor Intervention Project (STRIP): 6-year post-intervention follow-up. *Lancet Child Adolesc Health*. 2020;4(5):359-369. doi:10.1016/s2352-4642(20)30059-6

© 2023 Meng Y et al. *JAMA Pediatrics.*

16. Niinikoski H, Pahkala K, Ala-Korpela M, et al. Effect of repeated dietary counseling on serum lipoproteins from infancy to adulthood. *Pediatrics*. 2012;129(3):e704-13. doi:10.1542/peds.2011-1503

17. Friedewald WT, Levy RI, Fredrickson DS. Estimation of the concentration of low-density lipoprotein cholesterol in plasma, without use of the preparative ultracentrifuge. *Clin Chem*. Jun 1972;18(6):499-502.

18. Laitinen TT, Nuotio J, Juonala M, et al. Success in achieving the targets of the 20-year infancy-onset dietary intervention: Association with insulin sensitivity and serum lipids. *Diabetes Care*. 2018;41(10):2236-2244. doi:10.2337/dc18-0869

19. Pahkala K, Hietalampi H, Laitinen TT, et al. Ideal cardiovascular health in adolescence: Effect of lifestyle intervention and association with vascular intima-media thickness and elasticity (the Special Turku Coronary Risk Factor Intervention Project for Children [STRIP] study). *Circulation*. 2013;127(21):2088-96. doi:10.1161/circulationaha.112.000761

20. Pahkala K, Heinonen OJ, Simell O, et al. Association of physical activity with vascular endothelial function and intima-media thickness. *Circulation*. 2011;124(18):1956-1963. doi:10.1161/circulationaha.111.043851

21. Sankilampi U, Hannila M-L, Saari A, Gissler M, Dunkel L. New population-based references for birth weight, length, and head circumference in singletons and twins from 23 to 43 gestation weeks. *Ann Med*. 2013;45(5-6):446-454. doi:10.3109/07853890.2013.803739

22. Williams B, Mancia G, Spiering W, et al. 2018 ESC/ESH Guidelines for the management of arterial hypertension. *Eur Heart J*. 2018;39(33):3021-3104. doi:10.1093/eurheartj/ehy339

23. Buscot MJ, Magnussen CG, Juonala M, et al. The combined effect of common genetic risk variants on circulating lipoproteins is evident in childhood: A longitudinal analysis of the Cardiovascular Risk in Young Finns Study. *PLoS One*. 2016;11(1):e0146081. doi:10.1371/journal.pone.0146081

24. Madathil S, Blaser C, Nicolau B, Richard H, Parent ME. Disadvantageous socioeconomic position at specific life periods may contribute to prostate cancer risk and aggressiveness. *Front Oncol*. 2018;8:515. doi:10.3389/fonc.2018.00515

25. Madathil S, Joseph L, Hardy R, Rousseau MC, Nicolau B. A Bayesian approach to investigate life course hypotheses involving continuous exposures. *Int J Epidemiol*. 2018;47(5):1623-1635. doi:10.1093/ije/dyy107

26. Chumbley J, Xu W, Potente C, Harris KM, Shanahan M. A Bayesian approach to comparing common models of life-course epidemiology. *Int J Epidemiol*. 2021;50(5):1660-1670. doi:10.1093/ije/dyab073

27. Carpenter B, Gelman A, Hoffman MD, et al. Stan: A probabilistic programming language. *J Stat Softw*. 2017;76(1):1-32. doi:10.18637/jss.v076.i01

28. Raitakari OT, Juonala M, Rönnemaa T, et al. Cohort profile: The Cardiovascular Risk in Young Finns Study. *Int J Epidemiol*. Dec 2008;37(6):1220-6. doi:10.1093/ije/dym225

29. Akerblom HK, Viikari J, Uhari M, et al. Atherosclerosis precursors in Finnish children and adolescents. I. General description of the cross-sectional study of 1980, and an account of the children's and families' state of health. *Acta Paediatr Scand Suppl*. 1985;318:49-63. doi:10.1111/j.1651-2227.1985.tb10082.x

30. Armstrong MK, Fraser BJ, Hartiala O, et al. Association of non-high-density lipoprotein cholesterol measured in adolescence, young adulthood, and mid-adulthood with coronary artery calcification measured in mid-adulthood. *JAMA Cardiol*. 2021;6(6):661-668. doi:10.1001/jamacardio.2020.7238

© 2023 Meng Y et al. *JAMA Pediatrics.*

31. Meng Y, Buscot MJ, Juonala M, et al. Relative contribution of blood pressure in childhood, young- and mid-adulthood to large artery stiffness in mid-adulthood. *J Am Heart Assoc*. 2022;11(12):e024394. doi:10.1161/jaha.121.024394
32. Raitakari OT, Juonala M, Kähönen M, et al. Cardiovascular risk factors in childhood and carotid artery intima-media thickness in adulthood. *JAMA*. 2003;290(17):2277-2283. doi:10.1001/jama.290.17.2277
33. Juonala M, Viikari JS, Kähönen M, et al. Alcohol consumption is directly associated with carotid intima-media thickness in Finnish young adults: The Cardiovascular Risk in Young Finns Study. *Atherosclerosis*. Jun 2009;204(2):e93-98. doi:10.1016/j.atherosclerosis.2008.11.021
34. Rovio SP, Yang X, Kankaanpää A, et al. Longitudinal physical activity trajectories from childhood to adulthood and their determinants: The Young Finns Study. *Scand J Med Sci Sports*. Mar 2018;28(3):1073-1083. doi:10.1111/sms.12988
35. Kivimäki M, Vahtera J, Tabák AG, et al. Neighbourhood socioeconomic disadvantage, risk factors, and diabetes from childhood to middle age in the Young Finns Study: A cohort study. *Lancet Public Health*. Aug 2018;3(8):e365-e373. doi:10.1016/s2468-2667(18)30111-7

© 2023 Meng Y et al. *JAMA Pediatrics.*

eTable 1. Definitions of blood pressure classifications

Classifications	For life stage of infancy, preschool childhood, childhood[a]			For life stage of adolescence, young adulthood[b]		
	SBP	DBP	BP[c]	SBP	DBP	BP
Normal	SBP<90th percentile	DBP<90th percentile	SBP/DBP<90th percentile	SBP<120 mmHg	DBP<80 mmHg[d]	SBP/DBP <120/80 mmHg
Elevated	SBP ≥90th to <95th; or SBP≥ 120 mmHg to<95th percentile	DBP ≥90th to <95th; or DBP≥ 80 mmHg to<95th percentile	SBP/DBP ≥90th percentile to <95th percentile; or 120/80 mm Hg to <95th percentile	SBP:120-129 mmHg		SBP/DBP: 120/**<**80 to 129/**<**80 mmHg
Hypertension	SBP ≥95th percentile or ≥130 mmHg	DBP ≥95th percentile or ≥80 mmHg	SBP/DBP ≥95th percentile; or ≥130/80 mmHg	SBP≥130 mmHg	DBP≥80 mmHg	SBP/DBP≥ 130/80 mmHg

[a] Blood pressure classification was defined according to the 2017 American Academy of Pediatrics guidelines.
[b] Blood pressure classification was defined according to the 2018 American College of Cardiology/American Heart Association guidelines.
[c] Defined by systolic and diastolic blood pressure together.
[d] Both normal and elevated diastolic blood pressure were defined as <80mmHg.

© 2023 Meng Y et al. *JAMA Pediatrics.*

eTable 2. Systolic blood pressure levels at each age point, by intervention and sex.

Life-stages	Ages	Intervention				Control			
		Males		Females		Males		Females	
		Mean (SD), mmHg	Median (IQR), mmHg	Mean (SD), mmHg	Median (IQR), mmHg	Mean (SD), mmHg	Median (IQR), mmHg	Mean (SD), mmHg	Median (IQR), mmHg
Infancy		93 (12)	92 (86, 99)	91 (11)	91 (85, 98)	95 (9)	95 (90, 99)	93 (10)	93 (87, 98)
	7 months	91 (15)	93 (85, 99)	91 (15)	91 (83, 99)	93 (11)	94 (88, 100)	90 (13)	92 (85, 98)
	13 months	95 (13)	94 (86, 101)	91 (14)	92 (85, 99)	97 (12)	96 (90, 104)	96 (13)	96 (87, 103)
Preschool		98 (6)	98 (94, 102)	97 (8)	97 (93, 100)	100 (7)	99 (95, 104)	98 (7)	98 (93, 103)
	2 years	99 (10)	99 (93, 106)	96 (10)	95 (91, 100)	100 (17)	98 (92, 107)	97 (9)	97 (92, 103)
	3 years	98 (8)	98 (93, 104)	98 (10)	97 (92, 103)	101 (8)	101 (96, 105)	99 (9)	98 (94, 104)
	4 years	99 (8)	98 (95, 104)	99 (8)	98 (94, 103)	102 (9)	101 (96, 108)	99 (9)	100 (94, 105)
	5 years	96 (8)	96 (92, 102)	96 (9)	96 (91, 101)	96 (8)	95 (91, 100)	97 (9)	98 (91, 103)
Childhood		102 (5)	102 (99, 106)	103 (7)	102 (98, 106)	102 (6)	102 (98, 106)	103 (6)	103 (98, 107)
	6 years	100 (8)	99 (96, 104)	100 (8)	100 (95, 103)	99 (8)	99 (95, 103)	100 (7)	100 (96, 105)
	7 years	100 (7)	99 (96, 104)	100 (9)	100 (95, 104)	99 (7)	99 (95, 102)	102 (9)	101 (96, 107)
	8 years	99 (6)	99 (95, 103)	100 (8)	99 (95, 104)	100 (7)	100 (95, 104)	101 (7)	101 (96, 106)
	9 years	101 (6)	101 (98, 105)	102 (7)	102 (97, 106)	100 (6)	101 (96, 105)	101 (8)	101 (96, 106)
	10 years	104 (7)	104 (100, 109)	104 (9)	104 (99, 109)	106 (8)	105 (101, 110)	104 (9)	105 (100, 109)
	11 years	106 (7)	106 (101, 111)	107 (8)	105 (102, 110)	105 (8)	105 (99, 109)	106 (8)	106 (101, 110)
	12 years	108 (7)	106 (103, 112)	107 (8)	107 (102, 110)	108 (8)	107 (102, 112)	107 (8)	107 (102, 111)
Adolescence		117 (8)	116 (112, 120)	111 (6)	110 (107, 114)	117 (9)	116 (112, 120)	111 (8)	110 (106, 116)
	13 years	111 (8)	109 (105, 115)	108 (8)	108 (104, 111)	109 (8)	108 (105, 113)	109 (9)	107 (103, 113)
	14 years	114 (9)	112 (108, 118)	111 (7)	110 (107, 115)	115 (10)	113 (108, 120)	112 (9)	111 (106, 117)
	15 years	119 (10)	118 (113, 123)	112 (7)	111 (107, 115)	118 (10)	117 (111, 123)	112 (9)	111 (106, 117)
	16 years	119 (9)	118 (113, 123)	111 (7)	110 (107, 114)	120 (9)	118 (113, 126)	111 (9)	111 (106, 116)
	17 years	122 (10)	121 (116, 127)	113 (7)	112 (109, 116)	123 (9)	121 (114, 131)	112 (9)	112 (107, 117)
Young adulthood		126 (9)	126 (121, 132)	115 (7)	115 (111, 120)	126 (9)	125 (120, 130)	114 (9)	113 (110, 119)
	18 years	124 (9)	123 (119, 129)	115 (8)	114 (111, 118)	124 (9)	124 (117, 140)	113 (9)	113 (107, 118)
	19 years	126 (9)	125 (121, 131)	116 (7)	115 (110, 120)	127 (9)	125 (119, 132)	115 (9)	114 (109, 119)
	20 years	127 (9)	127 (123, 132)	116 (8)	115 (110, 120)	128 (10)	127 (122, 134)	115 (10)	115 (109, 120)
	26 years	127 (9)	127 (119, 134)	116 (7)	116 (111, 121)	125 (9)	125 (118, 131)	116 (9)	114 (109, 120)

Abbreviations: IQR, interquartile range; SD, standard deviation.

© 2023 Meng Y et al. *JAMA Pediatrics.*

eTable 3. Proportion, mean, and standard deviation of systolic blood pressure levels observed and derived[a].

Ages	Observed		Derived	
	% (n)	Mean, mmHg (Standard deviation)	% (n)[b]	Mean, mmHg (Standard deviation)
7 months	95 (1059)	93 (14)	5 (57)	94 (6)
13 months	86 (961)	95 (15)	14 (155)	95 (6)
2 years	79 (880)	99 (13)	21 (236)	98 (5)
3 years	74 (829)	100 (10)	26 (287)	99 (5)
4 years	73 (824)	101 (10)	26 (292)	100 (5)
5 years	70 (779)	96 (10)	30 (337)	100 (4)
6 years	67 (753)	100 (9)	33 (363)	100 (4)
7 years	63 (708)	101 (9)	37 (408)	101 (4)
8 years	60 (671)	100 (8)	40 (445)	101 (4)
9 years	55 (616)	101 (8)	45 (500)	103 (4)
10 years	55 (619)	104 (9)	45 (497)	104 (4)
11 years	54 (607)	106 (10)	46 (509)	107 (4)
12 years	53 (588)	107 (10)	47 (528)	108 (4)
13 years	52 (580)	109 (10)	48 (536)	110 (5)
14 years	50 (562)	114 (11)	50 (554)	112 (5)
15 years	50 (558)	117 (12)	50 (558)	114 (5)
16 years	48 (533)	116 (12)	52 (583)	116 (6)
17 years	46 (515)	118 (13)	54 (601)	118 (6)
18 years	45 (499)	119 (13)	55 (617)	120 (7)
19 years	43 (476)	121 (13)	57 (640)	122 (7)
20 years	42 (465)	121 (13)	58 (651)	123 (8)
26 years	49 (546)	121 (11)	51 (570)	123 (9)

[a] From the individual growth curve model.
[b] For each age, the proportion of missing systolic blood pressure values at that point is the proportion of systolic blood pressure values derived from the individual growth curve model.

© 2023 Meng Y et al. *JAMA Pediatrics.*

eTable 4. **Relative weights of systolic blood pressure measured at each individual age from 7 months to 26 years and carotid intima-media thickness measured at age of 26 years.**

Life-stages	Ages	Estimates are based on a per 1-SD[a] higher systolic blood pressure		Estimates are based on a per 10 mmHg higher systolic blood pressure	
		Relative weight, %	95% credible interval	Relative weight, %	95% credible interval
Infancy					
	7 months	7.6	0.2, 21.3	7.3	0.4, 18.1
	13 months	5.6	0.2, 17.4	4.9	0.2, 14.7
Preschool					
	2 years	3.1	0.1, 10.7	2.5	0.1, 8.9
	3 years	10.0	0.4, 26.8	9.9	0.4, 26.0
	4 years	4.9	0.2, 16.0	4.7	0.1, 15.3
	5 years	5.2	0.2, 17.0	5.1	0.1, 16.6
Childhood					
	6 years	5.5	0.2, 17.9	5.5	0.2, 17.9
	7 years	3.3	0.1, 11.5	3.4	0.1, 11.8
	8 years	4.0	0.1, 13.6	4.1	0.1, 14.3
	9 years	4.1	0.1, 14.0	4.4	0.1, 15.0
	10 years	4.0	0.1, 13.9	4.2	0.1, 14.3
	11 years	3.3	0.1, 11.5	3.4	0.1, 12.1
	12 years	4.5	0.1, 15.1	4.8	0.1, 15.9
Adolescence					
	13 years	4.2	0.1, 14.4	4.4	0.1, 14.8
	14 years	3.3	0.1, 11.5	3.4	0.1, 12.0
	15 years	3.0	0.1, 10.7	3.0	0.1, 10.4
	16 years	3.8	0.1, 12.9	4.0	0.1, 13.7
	17 years	4.5	0.1, 15.0	4.5	0.1, 14.9
Young adulthood					
	18 years	4.2	0.1, 14.3	4.1	0.1, 14.0
	19 years	3.9	0.1, 13.6	4.1	0.1, 14.0
	20 years	4.4	0.1, 15.0	4.5	0.1, 15.0
	26 years	3.6	0.1, 12.3	3.8	0.1, 12.9

Models adjusted for sex, height, smoking, body mass index, education levels, low-density lipoprotein cholesterol, high-density lipoprotein cholesterol, triglycerides, physical activity, birth weight for gestational age.

[a] Estimates are based on a per 1-SD (at each individual age-point) higher systolic blood pressure level at each age.

[b] Life stage averages for relative weights were calculated using mean values at 7 and 13 months for infancy, 2-5 years for preschool childhood, 6-12 years for childhood, 13-17 years for adolescence and 18-26 years for young adulthood.

© 2023 Meng Y et al. *JAMA Pediatrics.*

eTable 5. **Association of systolic blood pressure measured in infancy, preschool childhood, childhood, adolescence, and young adulthood and carotid intima-media thickness in young adulthood.**

Life-stages	Carotid intima-media thickness (N=349)			
	**β, mm**	**95%CrI**	**Relative weight, %**	**95%CrI**
Lifetime effect	0.02	0.01, 0.03		
Infancy			21.2	1.3, 47.7
Preschool childhood			31.8	2.8, 64.6
Childhood			17.8	0.7, 49.7
Adolescence			14.2	0.5, 42.3
Young adulthood			15.0	0.6, 42.7

Abbreviations: CrI, credible interval; N, number of participants.
β represents regression coefficient per 1-SD (at single life-stage) higher systolic blood pressure level. Estimates were derived from the model adjusted for intervention group, sex, height, smoking, body mass index, education levels, low-density lipoprotein cholesterol, high-density lipoprotein cholesterol, triglycerides, physical activity, birth weight for gestational age, blood glucose, anti-hypertensive medication use, alcohol consumption and parental history of hypertension.

© 2023 Meng Y et al. *JAMA Pediatrics.*

eTable 6. Association of systolic blood pressure measured in infancy, preschool childhood, childhood, adolescence, and young adulthood and carotid intima-media thickness in young adulthood.

Life-stages	Carotid intima-media thickness (N=341)			
	β, mm	95%CrI	Relative weight, %	95%CrI
Lifetime effect	0.02	0.01, 0.03		
Infancy			17.0	1.0, 41.9
Preschool childhood			35.7	3.5, 68.9
Childhood			18.5	0.7, 51.4
Adolescence			12.9	0.4, 39.9
Young adulthood			16.0	0.7, 44.9

Abbreviations: CrI, credible interval; N, number of participants.
β represents regression coefficient per 1-SD (at single life-stage) higher systolic blood pressure level. Estimates were derived from the model adjusted for intervention group, sex, height, smoking, body mass index, education levels, low-density lipoprotein cholesterol, high-density lipoprotein cholesterol, triglycerides, physical activity, birth weight for gestational age, blood glucose, anti-hypertensive medication use, alcohol consumption, parental history of hypertension, and types of feeding.

© 2023 Meng Y et al. *JAMA Pediatrics.*

eTable 7. Association of systolic blood pressure measured in infancy, preschool childhood, childhood, adolescence, and young adulthood and carotid intima-media thickness in young adulthood.

	Carotid intima-media thickness (N=341)			
**Life-stages**	**β, mm**	**95%CrI**	**Relative weight, %**	**95%CrI**
Lifetime effect	0.02	0.01, 0.03		
Infancy			23.1	1.6, 51.3
Preschool childhood			29.2	2.1, 62.7
Childhood			17.3	0.7, 49.2
Adolescence			14.3	0.5, 43.1
Young adulthood			15.9	0.6, 45.3

Abbreviations: CrI, credible interval; N, number of participants.
β represents regression coefficient per 1-SD (at single life-stage) higher systolic blood pressure level. Estimates were derived from the model adjusted for intervention group, sex, height, smoking, body mass index, education levels, low-density lipoprotein cholesterol, high-density lipoprotein cholesterol, triglycerides, physical activity, birth weight for gestational age, blood glucose, anti-hypertensive medication use, alcohol consumption, parental history of hypertension, and breastfeeding duration.

© 2023 Meng Y et al. *JAMA Pediatrics.*

eTable 8. Association of systolic blood pressure measured in infancy, preschool childhood, childhood, adolescence, and young adulthood and carotid intima-media thickness in young adulthood, stratified by sex.

	β, mm	95%CrI	Relative weight, %	95%CrI
Males				
Lifetime effect	0.02	0.01, 0.03		
Infancy			21.8	1.1, 52.6
Preschool childhood			29.7	1.6, 67.3
Childhood			18.5	0.6, 54.1
Adolescence			15.9	0.5, 48.2
Young adulthood			14.1	0.5, 43.7
Females				
Lifetime effect	0.02	0.01, 0.03		
Infancy			24.1	2.0, 50.6
Preschool childhood			18.2	0.8, 48.0
Childhood			20.0	0.9, 53.0
Adolescence			15.1	0.5, 45.2
Young adulthood			22.7	1.3, 54.8

Abbreviations: CrI, credible interval.

β represents regression coefficient per 1-SD (at single life-stage) higher systolic blood pressure level. Estimates were derived from the model adjusted for intervention group, height, smoking, body mass index, education levels, low-density lipoprotein cholesterol, high-density lipoprotein cholesterol, triglycerides, physical activity, birth weight for gestational age.

© 2023 Meng Y et al. *JAMA Pediatrics.*

eTable 9. Association of systolic blood pressure in infancy, preschool childhood, childhood, adolescence and young adulthood with carotid intima-media thickness in young adulthood

Life-stages	β, mm	95% CrI	Relative weight, %	95%CrI
Lifetime effect	0.02	0.01, 0.03		
Infancy			20.2	3.2, 39.2
Preschool childhood			13.6	1.5, 51.9
Childhood			14.8	1.0, 54.8
Adolescence			12.0	0.6, 44.6
Young adulthood			12.6	1.0, 47.5

Abbreviations: CrI, credible interval.

β represents regression coefficient per 10 mmHg higher systolic blood pressure level in each life stage (infancy, preschool childhood, childhood, adolescence and young adulthood) and the sum of these across the entire observed life-course (lifetime effect). Model adjusted for intervention group, sex, height, smoking, body mass index, education levels, low-density lipoprotein cholesterol, high-density lipoprotein cholesterol, triglycerides, physical activity, and birth weight for gestational age.

© 2023 Meng Y et al. *JAMA Pediatrics.*

eTable 10. Association of <u>diastolic blood pressure, pulse pressure and mean arterial pressure</u> measured in infancy, preschool childhood, childhood, adolescence, and young adulthood and carotid intima-media thickness in young adulthood.

	β, mm	95%CrI	Relative weight, %	95%CrI
Diastolic blood pressure				
Lifetime effect	0.00003	-0.01, 0.01		
Infancy				
Preschool childhood				
Childhood				
Adolescence				
Young adulthood				
Pulse pressure				
Lifetime effect	0.03	0.02, 0.04		
Infancy			30.1	3.2, 37.3
Preschool childhood			31.9	8.5, 53.8
Childhood			13.1	1.0, 35.1
Adolescence			12.0	1.0, 32.3
Young adulthood			13.2	1.0, 32.1
Mean arterial pressure				
Lifetime effect	0.01	0.003, 0.02		
Infancy			25.4	1.4, 58.1
Preschool childhood			17.9	1.0, 52.3
Childhood			20.2	1.0, 57.7
Adolescence			16.6	1.0, 50.4
Young adulthood			19.8	1.0, 54.7

Abbreviations: CrI, credible interval.
β represents regression coefficient per 1-SD (at single life-stage) higher blood pressure level.
Estimates are from the model adjusted for model 2 covariates of intervention, sex, height, smoking, body mass index, education levels, low-density lipoprotein cholesterol, high-density lipoprotein cholesterol, triglycerides, physical activity, and birth wight for gestational age.

© 2023 Meng Y et al. *JAMA Pediatrics.*

eTable 11. Association of systolic blood pressure, mean arterial pressure, and pulse pressure[a] measured from age 7 months up until the age at which carotid intima-media thickness was measured.

Age-point when cIMT was measured	SBP		MAP		PP	
	β, mm	95% CrI	β, mm	95%CrI	β, mm	95%CrI
11	0.01	0.003, 0.02	0.005	-0.002, 0.01	0.01	0.007, 0.02
13	0.02	0.01, 0.03	0.01	-0.003, 0.02	0.01	0.004, 0.02
15	0.01	0.005, 0.02	0.005	-0.001, 0.01	0.015	0.01, 0.02
17	0.01	0.004, 0.02	0.01	-0.0003, 0.01	0.01	0.007, 0.02
19	0.01	0.004, 0.01	0.002	-0.004, 0.007	0.01	0.005, 0.02
26	0.02	0.01, 0.03	0.01	0.003, 0.02	0.03	0.02, 0.04

Abbreviations: cIMT, carotid intima-media thickness; CrI, credible interval; MA, mean arterial pressure; PP, pulse pressure; SBP, systolic blood pressure.
[a] We examined these three blood pressure components with cIMT at earlier age-points (11-19 years), because they associated with carotid intima-media thickness at ages of 26 years.
β represents regression coefficient for per 1-SD (at single age-point) higher pulse pressure level across the period from age 7 months to the age at which intima-media thickness was measured.
For intima-media thickness measured at age 13, 15, 17, 19, and 26 years, estimates are from the model adjusted for model 2 covariates of intervention, sex, height, smoking, body mass index, education levels, low-density lipoprotein cholesterol, high-density lipoprotein cholesterol, triglycerides, physical activity, and birth wight for gestational age. For intima-media thickness measured at age of 11, estimates were adjusted for the covariates as mentioned above other than physical activity and smoking given these two variables were first collected at age 13 years follow-up and thereafter.

© 2023 Meng Y et al. *JAMA Pediatrics.*

eTable 12. Baseline (age 7 months) characteristics of participants and non-participants at the age 26 years follow-up

Characteristics	Non-participants		Participants		P value[b]
	n	Statistic[a]	n	Statistic[a]	
Intervention, % (n)	582	53 (310)	534	48 (254)	0.06
Females, % (n)	582	42 (245)	534	56 (299)	0.001
Observed SBP, mmHg	551	93 (15)	508	93 (14)	0.86
Observed DBP, mmHg	551	59 (13)	508	59 (12)	0.64
Observed and derived[c] SBP, mmHg	582	94 (14)	534	93 (14)	0.63
Observed and derived[c] DBP, mmHg	582	58 (13)	534	58 (11)	0.64
Height, cm	580	70.5 (2.4)	526	70.2 (2.5)	0.38
Body mass index, kg/m^2	580	17.2 (1.5)	526	17.2 (1.4)	0.86
Non-HDL-C, mmol/L	437	3.17 (0.71)	402	3.23 (0.85)	0.63
HDL-C, mmol/L	437	0.93 (0.20)	402	0.91 (0.20)	0.23
Birth weight for gestational age, % (n)					0.18
Small		11 (62)		10 (49)	
Appropriate		81 (450)		80 (410)	
Large		8 (46)		10 (56)	
Parental history of hypertension, % (n)		34 (125)		32 (109)	0.17

Abbreviations: DBP, diastolic blood pressure; HDL-C, high-density lipoprotein cholesterol; SBP, systolic blood pressure.
[a] Mean (standard deviation) for continuous variables or n (proportions) for categorical variables
[b] From logistic regression analyses that compared participants and non-participants at follow-up. Because a higher proportion of females participated at follow-up, all subsequent comparisons were adjusted for sex.
[c] Observed blood pressure values incorporated with individual growth curve model-derived values.

© 2023 Meng Y et al. *JAMA Pediatrics.*

eTable 13. Association of systolic blood pressure measured in infancy, preschool childhood, childhood, adolescence, and young adulthood and carotid intima-media thickness in young adulthood.

	β, mm	95%CrI	Relative weight, %	95%CrI
Standard model				
Lifetime effect	0.02	0.01, 0.03		
Infancy			24.3	3.5, 46.2
Preschool childhood			28.7	3.2, 57.2
Childhood			13.7	0.5, 40.0
Adolescence			12.9	0.4, 37.5
Young adulthood			20.3	1.5, 46.0
Residual model				
Lifetime effect	0.02	0.01, 0.03		
Infancy			28.7	4.4, 53.0
Preschool childhood			23.5	1.6, 53.9
Childhood			10.7	0.3, 34.2
Adolescence			12.1	0.4, 37.6
Young adulthood			25.1	2.4, 53.6

Abbreviations: CrI, credible interval.
β represents regression coefficient per 1-SD (at single life-stage) higher systolic blood pressure level.
Standard model is adjusted for sex, intervention and lifetime averaged values for height and body mass index, in which the average of systolic blood pressure at each life stage was used as the primary exposure variable. Residual model is adjusted for sex, intervention and used the residual from a regression analysis at each life stage where systolic blood pressure was regressed on height and body mass index (i.e., with the form “systolic blood pressure ~ height + body mass index”).

© 2023 Meng Y et al. *JAMA Pediatrics.*

eTable 14. Systolic blood pressure at each observed time-point in the Cardiovascular Risk in Young Finns Study.

Years prior to the time point at which cIMT was measured (N=1865)	Mean, mmHg	Standard deviation, mmHg	Median, mmHg	interquartile range, mmHg
0 (aged 33−45 years)	121	14	119	110, 130
3 (aged 30−42 years)	119	10	118	111, 126
6 (aged 27−39 years)	117	13	116	108, 125
9 (aged 24−36 years)	118	9	118	111, 125
12 (aged 21−33 years)	119	9	118	112, 125
15 (aged 18−30 years)	118	9	119	112, 125
18 (aged 15−27 years)	118	8	118	112, 124
21 (aged 12−24 years)	117	11	116	109, 125
24 (aged 9−21 years)	116	11	115	108, 124
27 (aged 6−18 years)	113	11	112	106, 121

Abbreviation: cIMT, carotid intima-media thickness.

© 2023 Meng Y et al. *JAMA Pediatrics.*

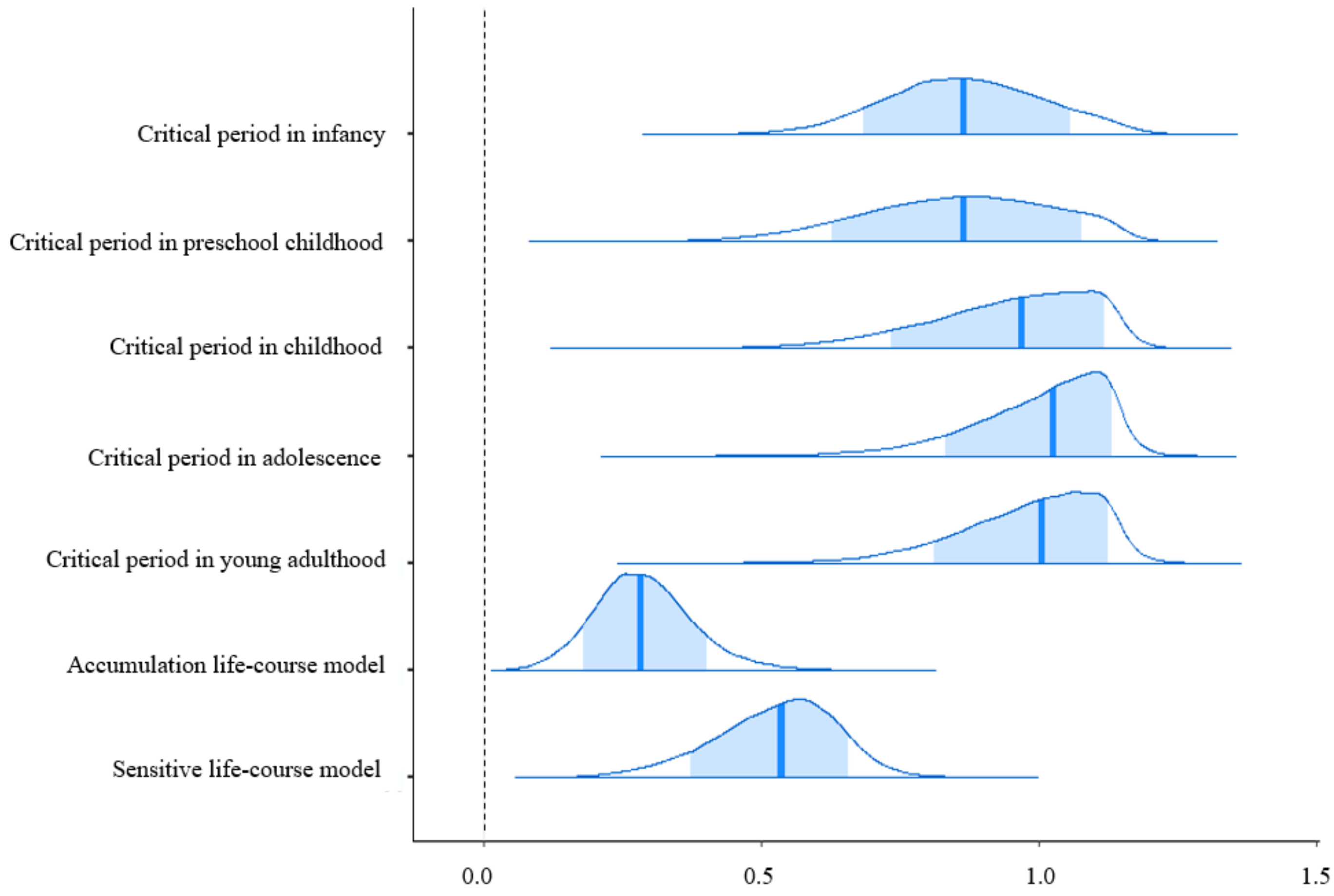

eFigure 1. Median and 80% credible intervals of posterior distributions of Euclidean distance under three life-course scenarios for the association of systolic blood pressure and carotid intima-media thickness.

The vertical solid line indicates the median, the grey area indicates the 80% credible interval. The Y axis shows reference vectors to estimated weights of: critical period in infancy (weight in infancy = 100%, weight in preschool childhood = 0, weight in childhood = 0, weight in adolescence = 0, weight in young adulthood = 0), critical period in preschool childhood (weight in infancy = 0, weight in preschool childhood = 100%, weight in childhood = 0, weight in adolescence = 0, weight in young adulthood = 0), critical period in childhood (weight in infancy = 0, weight in preschool childhood = 0, weight in childhood = 100%, weight in adolescence = 0, weight in young adulthood = 0), critical period in adolescence (weight in infancy = 0, weight in preschool childhood = 0, weight in childhood = 0, weight in adolescence = 100%, weight in young adulthood = 0), critical period in young adulthood (weight in infancy = 0, weight in preschool childhood = 0, weight in childhood = 0, weight in adolescence = 0, weight in young adulthood = 100%), sensitive life-course model (weight in infancy = 2.5%, weight in preschool childhood = 7.5%, weight in childhood = 12.5%, weight in adolescence = 20%, weight in young adulthood = 57.5%), accumulation life-course model (all weights =1/5).

© 2023 Meng Y et al. *JAMA Pediatrics.*

A. Trace plot

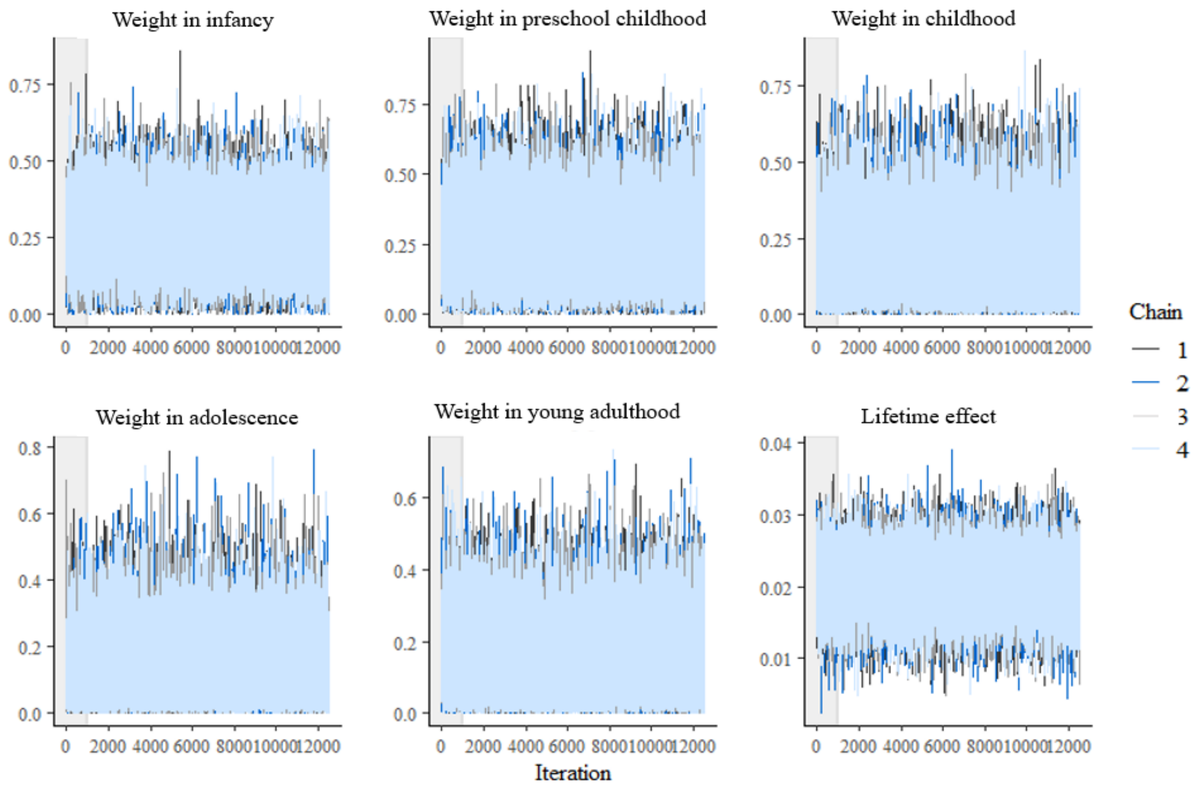

B. Rhat values

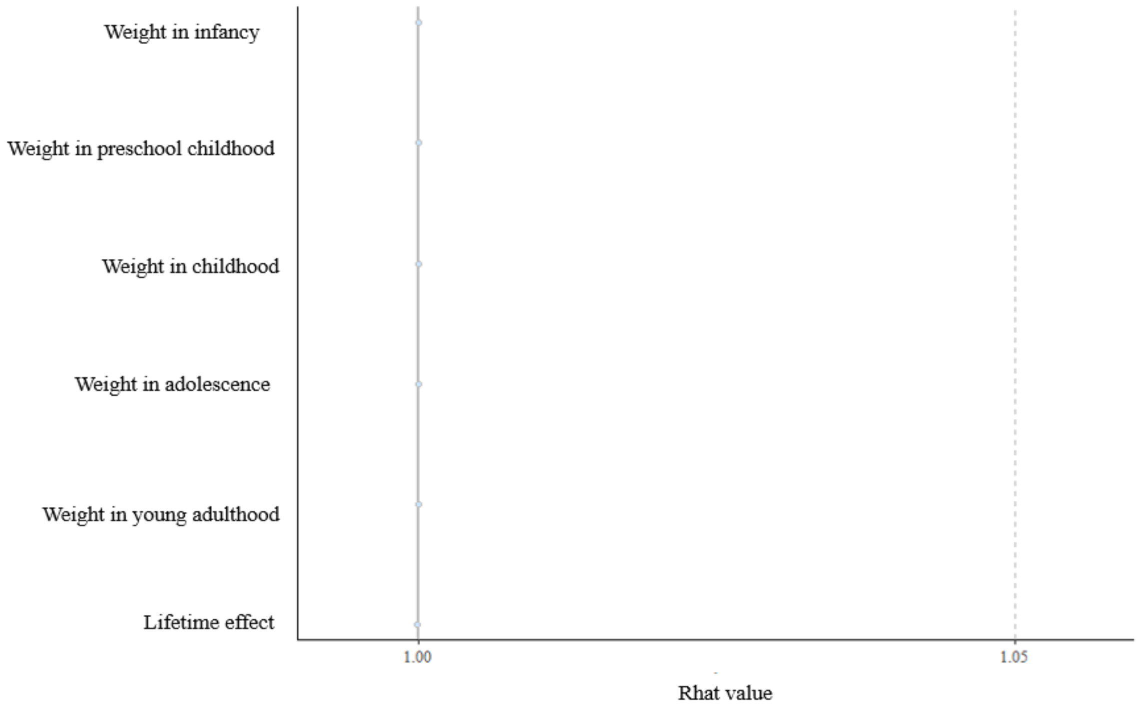

© 2023 Meng Y et al. *JAMA Pediatrics.*

C. Effective sample size

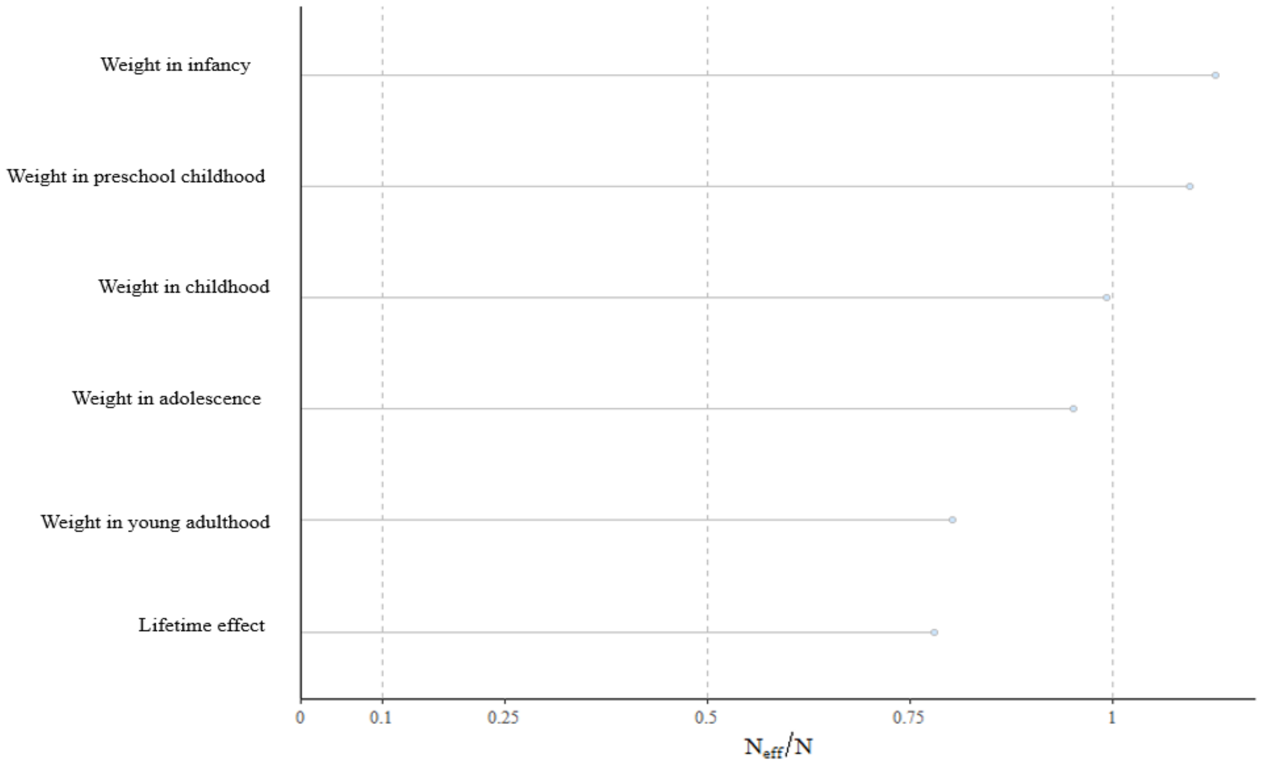

D. Autocorrelation function plots

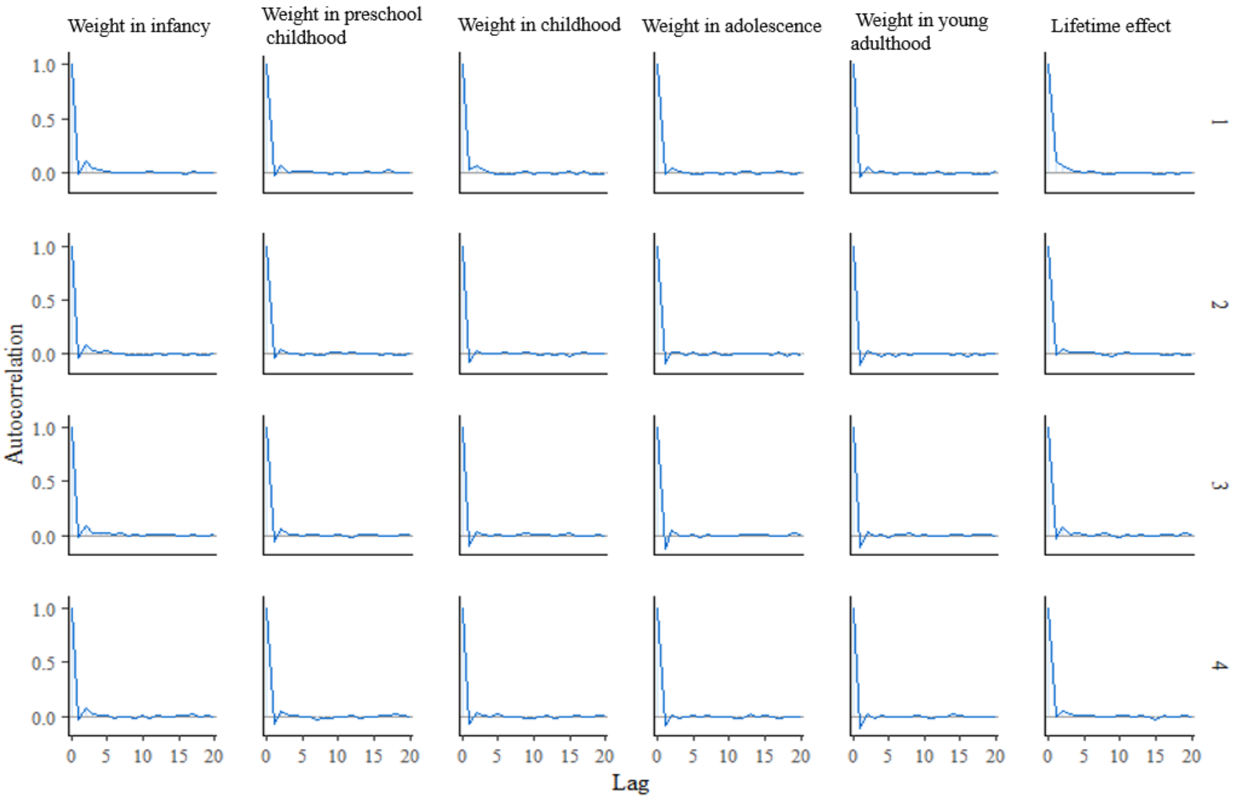

© 2023 Meng Y et al. *JAMA Pediatrics.*

E. Identifiability

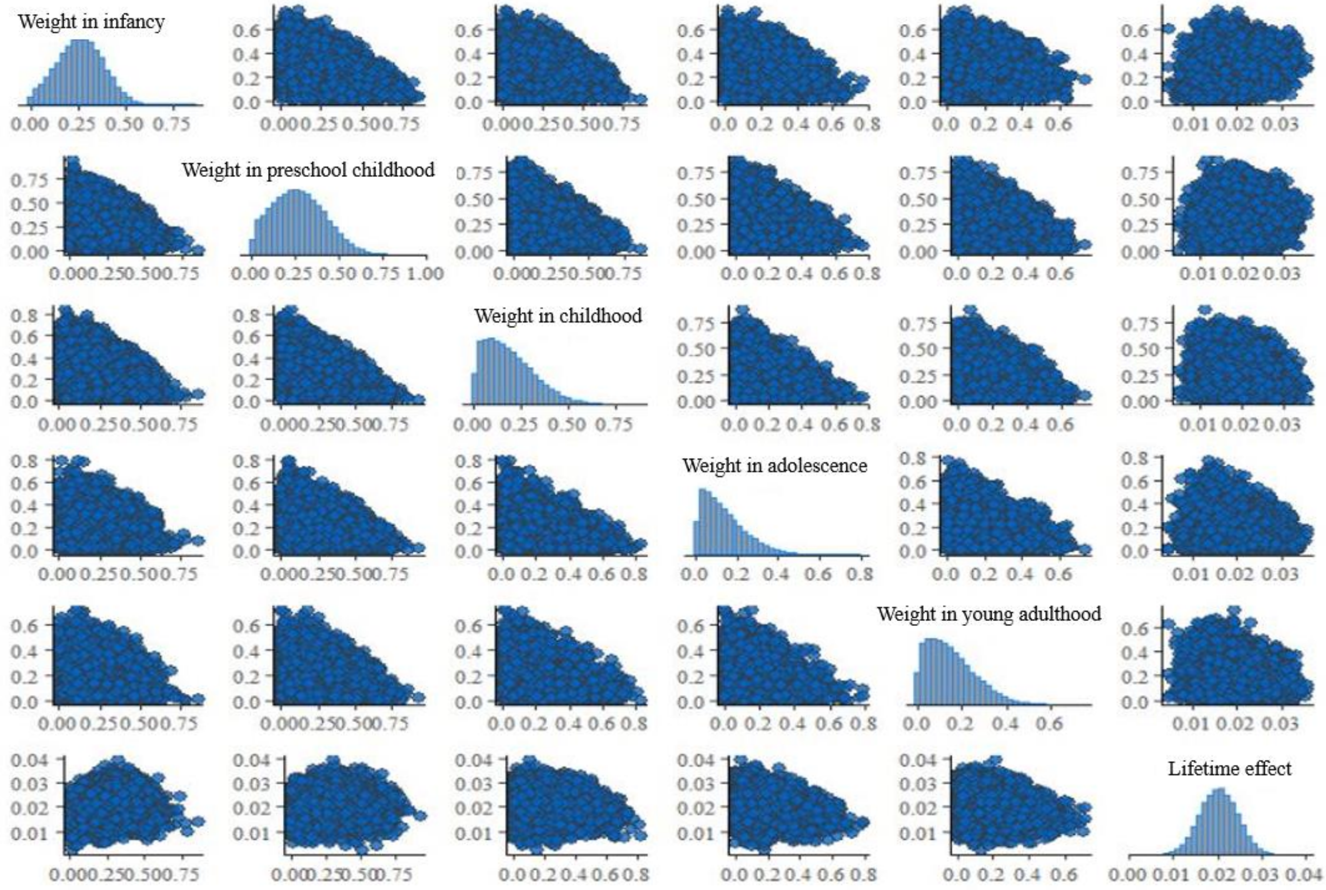

eFigure 2. Diagnostics of the Bayesian relevant life-course exposure model, including (A) trace plots, (B) Rhat values, (C) effective sample size, (D) autocorrelation function plots, (E) identifiability of the parameters, for systolic blood pressure and carotid intima-media thickness.

© 2023 Meng Y et al. *JAMA Pediatrics.*

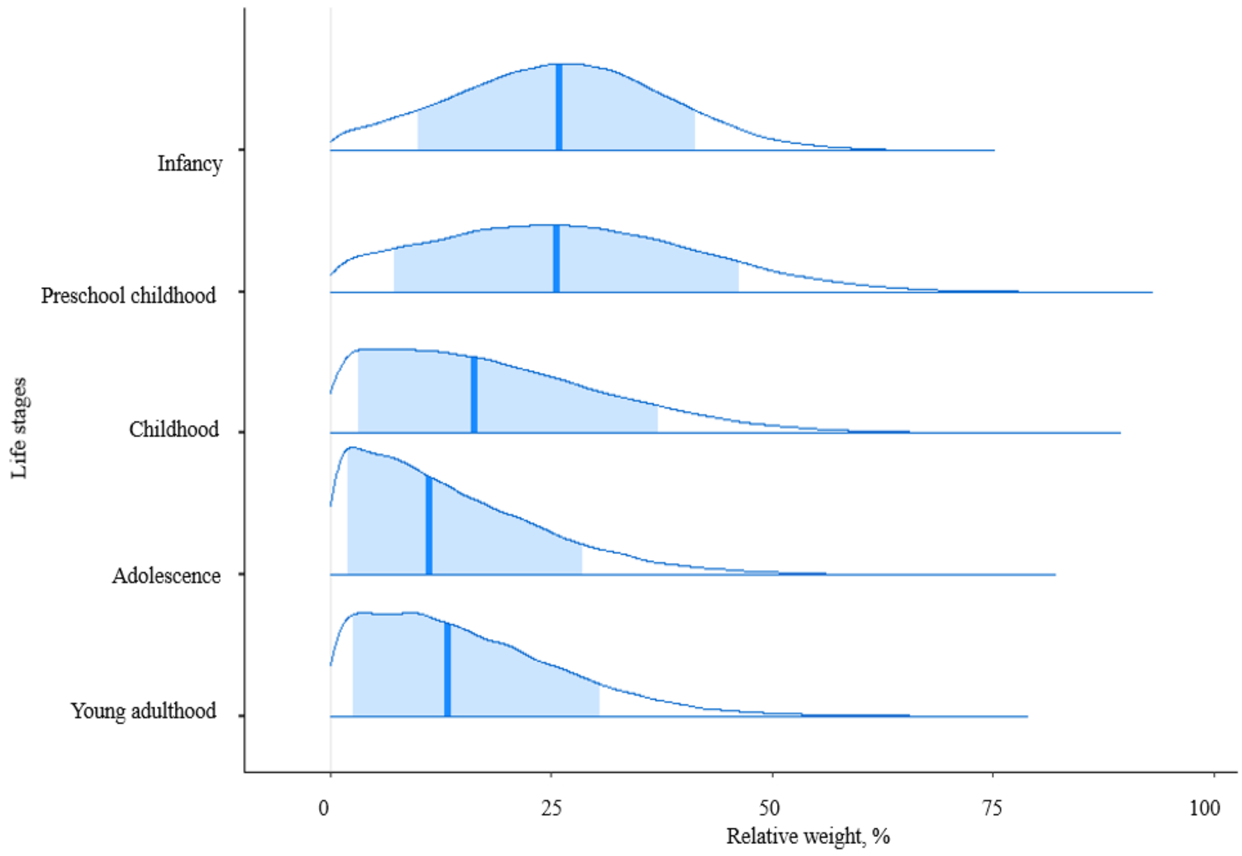

eFigure 3. Posterior densities of relative weights for exposure to systolic blood pressure in infancy, preschool childhood, childhood, adolescence, and young adulthood and carotid intima-media thickness in young adulthood.
The vertical solid line indicates the median, the blue area indicates the 80% credible interval.
Estimates from the model adjusted for model 2 covariates of intervention, sex, height, smoking, body mass index, education levels, low-density lipoprotein cholesterol, high-density lipoprotein cholesterol, triglycerides, physical activity, and birth wight for gestational age.

© 2023 Meng Y et al. *JAMA Pediatrics.*

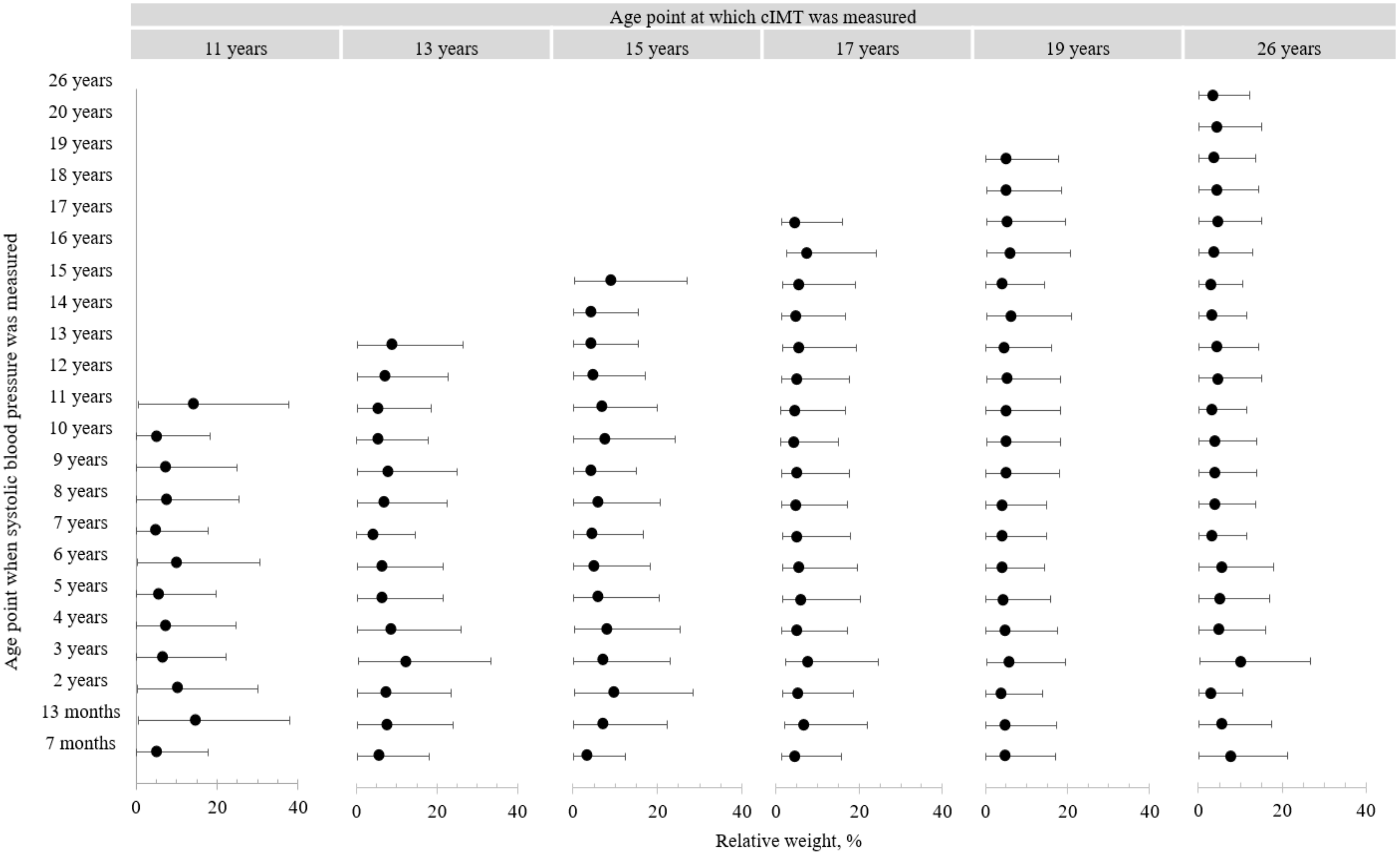

© 2023 Meng Y et al. *JAMA Pediatrics.*

eFigure 4. Relative weights of systolic blood pressure measured across each age prior to, and concurrent with, the age when carotid intima-media thickness was measured.

Dots and values represent relative weight; error bars represent 95% credible interval for intima-media thickness measured at age of 13, 15, 17, 19, and 26 years, estimates from the model adjusted for model 2 covariates of intervention, sex, height, smoking, body mass index, education levels, low-density lipoprotein cholesterol, high-density lipoprotein cholesterol, triglycerides, physical activity, and birth wight for gestational age. For intima-media thickness measured at age 11 years, estimates were adjusted for the covariates as mentioned above other than physical activity and smoking given these two variables were first collected at age 13 years follow-up and thereafter. Estimates are based on a per 1-SD (at individual age-point) higher systolic blood pressure level at each age.

© 2023 Meng Y et al. *JAMA Pediatrics.*

A. Model 1

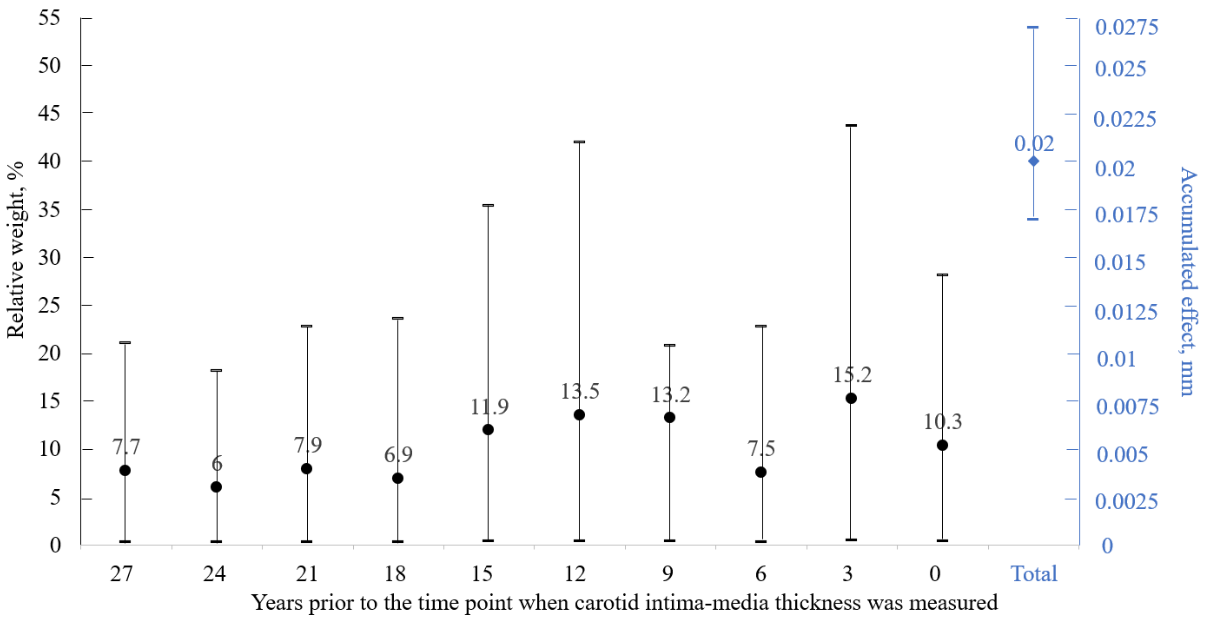

B. Model 2

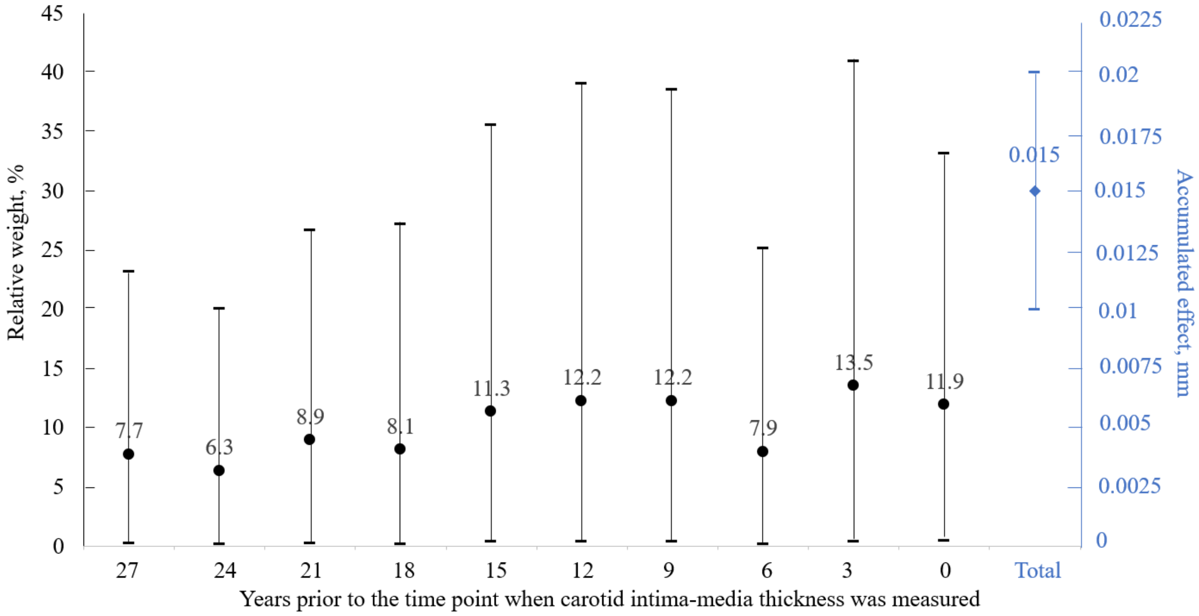

eFigure 5. Association of systolic blood pressure measured at multiple age points from 0 (aged 33 to 45 years) to 27 years (aged 6 to 18 years) prior to carotid intima media thickness measured in mid-adulthood (aged 33 to 45 years), using data from The Cardiovascular Risk in Young Finns Study.
Black dots and values represent relative weight point estimates. Blue diamond and value represent accumulated association per 1-SD (at individual age-point) higher systolic blood pressure across all observed time-points and carotid intima-media thickness in mid-adulthood (accumulated effect). Black error bars indicate the 95% credible interval of relative weights. Blue error bars indicate the 95% credible interval of the accumulated effect. Right Y axis represents the scale for the accumulated effect. Left Y axis represents the scale for the relative weight.
A) Model 1 adjusted for year of birth, sex and height. B) Model 2 adjusted for year of birth, sex, height, smoking, body mass index, education levels, low-density lipoprotein cholesterol, high-density lipoprotein cholesterol, triglycerides, and physical activity index.

© 2023 Meng Y et al. *JAMA Pediatrics.*
