# Supplementary material for: Blood Pressure at Different Life Stages Over the Early Life Course and Intima-Media Thickness
Source: JAMA Pediatr. 2023 Dec 4;178(2):133–41. doi: 10.1001/jamapediatrics.2023.5351 (PMC10696511; doi:10.1001/jamapediatrics.2023.5351)
Supplement: Supplement 2. — Data sharing statement [file jamapediatr-e235351-s002.pdf]

Data Sharing Statement

Meng. Blood Pressure at Different Life Stages Over the Early Life Course and Intima-Media Thickness. *JAMA Pediatr*. Published December 04, 2023.
doi:10.1001/jamapediatrics.2023.5351

Data
Data available: No

Additional Information
Explanation for why data not available: Data access may be permitted on a case-by-case basis upon request only. Investigators can submit an expression of interest to the chairman of the STRIP steering group (Prof. Olli Raitakari).
